# Phylogenetic Analysis of the Family Lepidostomatidae (Trichoptera: Integripalpia) Using Whole Mitochondrial Genomes

**DOI:** 10.3390/insects16050536

**Published:** 2025-05-19

**Authors:** Xinyu Ge, Jingyuan Wang, Zhen Deng, Lu Chai, Wei Cao, Wenbin Liu, Jiwei Zhang, Chuncai Yan

**Affiliations:** 1 Tianjin Key Laboratory of Conservation and Utilization of Animal Diversity, College of Life Sciences, Tianjin Normal University, Tianjin 300387, China; skygxy@tjnu.edu.cn (X.G.); 2310170022@stu.tjnu.edu.cn (J.W.); 2410170016@stu.tjnu.edu.cn (L.C.); caowei@stu.tjnu.edu.cn (W.C.); skylwb@tjnu.edu.cn (W.L.); 2 Department of Entomology, College of Plant Protection, Nanjing Agricultural University, Nanjing 210095, China; 2024202059@stu.njau.edu.cn; 3 Changjiang Basin Ecology and Environment Monitoring and Scientific Research Center, Changjiang Basin Ecology and Environment Administration, Ministry of Ecology and Environment, Wuhan 430010, China

**Keywords:** mitogenome, phylogeny, *Lepidostoma*, *Paraphlegopteryx*

## Abstract

Lepidostomatidae is a relatively large family of the infraorder Plenitentoria (Integripalpia), comprising four fossil genera and seven extant genera. Lepidostomatid adults exhibit pronounced sexual dimorphism and have thus been referred to as the ‘cabinet of curiosities’ within Trichoptera. However, only five annotated mitogenomes of *Lepidostoma* have been recorded in the GeneBank database, and some of these mitogenomes are incomplete. To better understand the structure of mitogenome and phylogenetic relationships of Lepidostomatidae, we present mitogenomes of 13 *Lepidostoma* species and one *Paraphlegopteryx* species for the first time. We combined these new mitogenomes with previously published data for a comparative analysis. The results showed that the structure of mitogenome was relatively conserved, the nucleotide composition was significantly AT biased, and the control region showed the highest A + T content. Evolutionary rate analysis showed that all protein-coding genes underwent purification selection. The phylogenetic relationships supported the monophyly of Lepidostomatidae and restored the taxonomic positions of the two subfamilies. Meanwhile, two monophyletic branches (*Lepidostoma ferox* branch and *Lepidostoma hirtum* branch) within the genus *Lepidostoma* were also strongly supported. These findings significantly advance our understanding of the mitogenome and phylogeny of Lepidostomatidae.

## Simple Summary

This study sought to clarify the fundamental characteristics and phylogenetic relationships of the mitochondrial genome within the family Lepidostomatidae. We have for the first time reported the mitochondrial genomes of 13 species of the genus *Lepidostoma* and 1 species of the genus *Paraphlegopteryx*. Through a comprehensive comparative genomic analysis of Lepidostomatidae, we systematically studied their mitogenome base composition, codon usage, and evolutionary rate. Furthermore, we integrated all available lepidostomatid mitogenomes and reconstructed their phylogenetic relationships using maximum likelihood and Bayesian inference methods. This study significantly increased the mitogenomic data of Lepidostomatidae, providing a scientific basis for the identification of females and larvae of Lepidostomatidae and offering new insights into the phylogenetic relationships within the family Lepidostomatidae.

## 1. Introduction

Well known for their diversity and abundance, Trichoptera is a prominent group of insects inhabiting various global aquatic ecosystems and is extensively utilized for assessing the impact of pollutants within these systems [1,2]. Ulmer first described Lepidostomidae as a subfamily of Sericostomidae [3]. The family Lepidostomatidae is divided into two subfamilies (Lepidostomatinae Ulmer, 1903 and Theliopsychinae Weaver, 1993), including four fossil genera and seven extant genera. Among them, four fossil genera were reported from the Baltic region (†*Archaeocrunoecia* Ulmer, 1912, †*Electrocrunoecia* Ulmer, 1912 and †*Maniconeurodes* Ulmer, 1912) and United Kingdom (†*Eucrunoecia* Sukatsheva & Jarzemboski, 2001), respectively [4]. In seven extant genera, the genus *Lepidostoma* Rambur, 1842 is the largest genus of Lepidostomatidae, exhibiting a cosmopolitan distribution. In contrast, the genus *Hummeliella* Forsslund, 1936 were merely reported from China [5]. The genus *Paraphlegopteryx* Ulmer, 1907 and the genus *Zephyropsyche* Weaver, 1993 were reported from the East Palearctic region and Oriental region. The genus *Theliopsyche* Banks, 1911, *Martynomyia* Fischer, 1970, and the genus *Crunoecia* McLachlan, 1876 were reported from Nearctic region and West Palearctic region, respectively.

**Figure 1:**
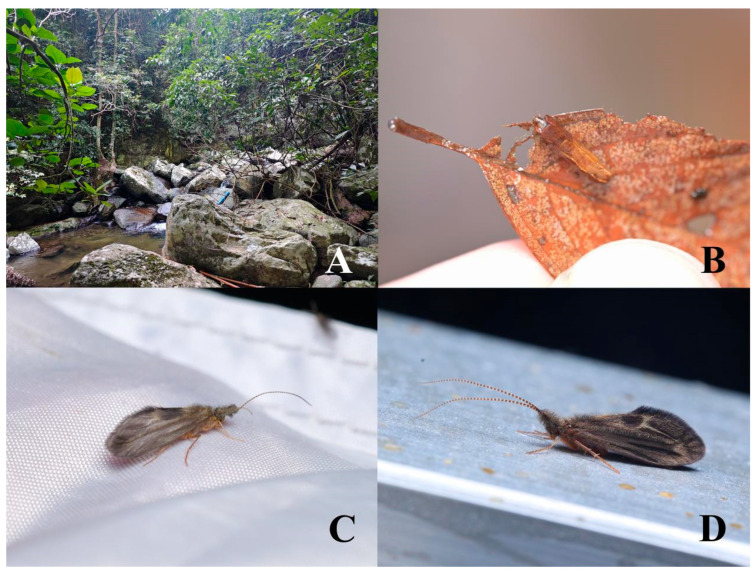
(**A**) Habitat of Lepidostomatidae; (**B**) larva of the genus *Lepidostoma*; (**C**) *Lepidostoma sichuanense*, male, from Sichuan, China, (photographed by Wei Cao); (**D**) *Lepidostoma longipilosum*, male, from Hebei, China, (photographed by Qianle Lu).

The lepidostomatid larvae most often occur in cool streams (Figure 1A), usually in low-flow areas, but they are also found in lakes and in temporary pools [6,7,8,9]. Larvae generally have a univoltine life cycle and use dead plant material and grit to construct portable tubular cases [10,11]. Most early instar larvae use grit to build cylindrical cases, while late-instar larvae construct quadrangular cases made of square pieces of bark or leaves attached at the front (Figure 1B) [12,13]. These larvae are primarily detritivorous, feeding on decomposing organic matter within their habitat [14]. The lepidostomatid adult exhibits striking sexual dimorphism, especially adult males, of the maxillary palps, antennal scapes, wings, and forelegs, and is therefore also known as the “cabinet of curiosities” within the order Trichoptera [15,16]. Adult males of different *Lepidostoma* species exhibit pronounced variation in antennal scape morphology and maxillary palp structure (Figure 1C,D) [17]. The secondary sexual characteristics of this genus are highly specialized, which provides an important basis for identifying closely related species with similar genital structures. In contrast, adult females and larvae show minimal interspecific differentiation in morphological traits, posing significant challenges for species-level identification.

At present, accurate species identification and the determination of phylogenetic relationships among some genera within Lepidostomatidae still are challenges. Previous studies on this family have mainly focused on morphological analyses. For instance, Weaver presented a synopsis of the North American Lepidostomatidae species and later synonymized the caddisflies of the genus *Lepidostoma* [15,18]. Additionally, larval stages of this family have been extensively documented in stream ecology studies due to their ecological significance [19]. However, molecular data within the family Lepidostomatidae are severely lacking [20].

Mitochondrial genomes (mitogenomes) generally contain 13 protein-coding genes (PCGs), 22 transfer RNAs (tRNAs), two ribosomal RNAs (rRNAs), and one or more control regions (CRs) [21,22]. Because of the importance of cellular energy production, a mitogenome exhibits strong evolutionary conservation in both gene content and arrangement [23]. The full-length mitogenome sequence can be more phylogenetically informative than the shorter sequences of individual molecular markers, which has been widely used for inferring phylogenetic relationship and species identification within Trichoptera at different taxonomic levels [24,25,26]. In Trichoptera, a large number of mitochondrial genome data have been published in the last two years, and these data have contributed significantly to species classification, larval matching, phylogenetic indications, and environmental DNA metabarcoding technology for water quality monitoring [2]. However, only five annotated mitogenomes of *Lepidostoma* species have been recorded in the NCBI database. Compared with other families of the suborder Integripalpia, mitogenomes of Lepidostomatidae are relatively rare, especially the Chinese species and the mitochondrial gene order and base composition of other genera within the Lepidostomatidae remain unknown. This limits research on the classification and phylogeny of the family.

In this study, 14 lepidostomatid species of mitogenomes were assembled and annotated; among them, seven species have never had any molecular data reported. Notably, this work presents the first mitogenome report for the genus *Paraphlegopteryx*. In addition, we also annotated the published mitogenome of *Crunoecia irrorata* Curtis, 1834. Combination with published, partial mitogenomes of Lepidostomatidae (including three species from GenBank), we analyzed the features of the mitogenomes, including nucleotide composition, and evolutionary rates. Subsequently, a phylogeny of the genus level within the Lepidostomatidae was reestablished using maximum likelihood (ML) and Bayesian inference (BI) models. This study not only enriches the mitogenome database of Lepidostomatidae but also provides understanding of the lepidostomatid phylogeny. The results will facilitate robust molecular identification of females and larvae and support the application of environmental DNA (eDNA) metabarcoding for biodiversity monitoring and conservation efforts.

## 2. Materials and Methods

### 2.1. Taxon Sampling and DNA Extraction

A total of 14 species from Lepidostomatidae were collected using Malaise traps, light traps (with a white tent and 250 W high-pressure mercury lamp), or pan traps (with 15 W ultraviolet light tubes) in China between July 2022 and August 2024 (Supplementary Table S1). All specimens were preserved in 100% ethanol and stored at −70 °C before DNA extraction. Morphological identification of the samples was conducted for Xinyu Ge by diagnostic keys of Yang and Weaver [17]. Genomic DNA was extracted from the legs of the specimen, using the TIANamp Genomic DNA Kit (DP304; TIANGEN, Beijing, China). Specimen vouchers and surplus DNA from each sequenced species have been deposited in the College of Life Science, Tianjin normal University, Tianjin, China.

### 2.2. Sequencing, Assembly, and Annotation

Genomic sequencing was performed at Novogene Co., Ltd. (Tianjin, China), using the Illumina NovaSeq x plus platform (Illumina Company, San Diego, CA, USA) and the DNBSEQ-T7 platform (Beijing Genomics Institution, Beijing, China), with 350 bp inserts and a paired-end 150 bp reads sequencing strategy. About 6 Gb (10× coverage per sample) of clean data was obtained for each sample. De novo assembly of mitogenomes used NOVOPlasty v3.8.3 (Brussel, Belgium) [27], with k-mer = 39 (default parameters), and with *mtCOI* as seed sequences. To check the accuracy of mitogenomes, we used IDBA-UD v1.1.3 (HongKong, China) [28] to independently assemble the genome and filtered out mitogenomes in Geneious v2025.2.1 (Boston, MA, USA) [29]. The raw mitogenome of *C. irrorata* was downloaded from NCBI. The tRNA genes were identified using the MITOS2 webserver [30], while PCGs and rRNA genes were annotated by alignment to related species in Geneious. All mitogenome sequences were deposited in GenBank (Table S2). Finally, the mitogenome maps were generated using CG-view (https://cgview.ca/, accessed on 30 January 2025).

### 2.3. Comparative Mitogenomic Analyses

Mitochondrial nucleotide composition and strand asymmetry were calculated with SeqKit v2.8.2 (Chongqing, China) [31]. The composition skew values were calculated by the following formulas: GC skew = G − C/G + C and AT skew = A − T/A + T [32]. The rates of the non-synonymous substitution rate (Ka)/synonymous substitution rate (Ks) and the nucleotide diversity for 13 PCG of the 14 lepidostomatid species were calculated using DnaSP v6.0. (Barcelona, Spain) [33]. The relative synonymous codon usage (RSCU) of the 14 lepidostomatid species were calculated and visualized using MEGA X (Philadelphia, PA, USA) and R v4.0.3 [34], respectively.

### 2.4. Phylogenetic Analyses

A total of 27 species were selected for phylogenetic analyses, 18 species as ingroups, and 9 species (seven genera) from the other four families (Phryganopsychidae Wiggins, 1959, Phryganeidae Leach, 1815, Pisuliidae Ross, 1967, Brachycentridae Ulmer, 1903) as outgroups, based on the phylogeny of Trichoptera (Table S2) [2]. Two rRNAs and 13 PCGs from each species were individually aligned using MAFFT v7.470 (Osaka, Japan) [35] (L-INS-I model) and trimmed using trimAl v1.4.1 (Barcelona, Spain) (default parameters) [36]. FASconCAT-G v1.05 (Bonn, Germany) [37] was applied to concatenate aligned sequences from each gene, and five datasets were generated. Five datasets were used for phylogenetic analyses: (1) PCG (all codon positions of the PCGs), (2) PCG12 (the 1st and 2nd codon positions of the PCGs), (3) PCG123R (all codon positions of the PCGs and rRNAs), (4) PCG12R (the 1st and 2nd codon positions of the PCGs and rRNAs), and (5) AA (amino acid sequences of the PCGs). The heterogeneity analysis of five datasets, using ALIGROOVE v1.0.7 [38] (Bonn, Germany) and DAMBE v.7.2.32 (Ottawa, ON, Canada) [39], was assessed to evaluate the substitution saturation of nucleotide datasets.

Phylogenetic relationship reconstruction was carried out with the ML and BI method. The best partitioning scheme and the best-fit substitution model for each partition were tested using ModelFinder [40]. ML analysis using IQ-TREE v 2.2.0.8 (Canberra, ACT, Australia) [41] used the best-fit substitution model and 1000 bootstrap replicates [42]. BI analysis was conducted using Phylobayes-MPI v1.9 (Montréal, QC, Canada) [43], run with MCMC (Markov chain Monte Carlo) analysis, and stopped after the two runs had satisfactorily converged (maxdiff < 0.3; effsize > 50). The initial 25% of trees were discarded as burn-in, and a strict consensus tree was generated from the remaining trees. Phylogenetic results are displayed in Figtree v1.4.3 (Oxford, UK) [44].

## 3. Results and Discussion

### 3.1. Mitogenome Features and Base Composition of Lepidostomatidae

**Figure 2:**
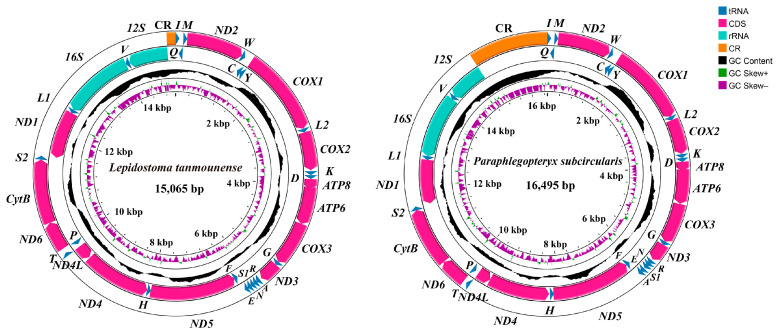
The mitogenome map of two lepidostomatid species. The arrow served as a guide, pointing to the orientation of gene transcription. We used standardized abbreviations to denote PCGs and rRNAs, while single-letter abbreviations were chosen for tRNAs.

We obtained novel mitogenomes for 14 species (two genera) from Lepidostomatidae, with *Paraphlegopteryx* reported for the first time (Figure 2, Figures S1 and S2). Newly sequenced mitogenomes contained the entire set of 37 genes (13 PCGs, 22 tRNA genes, and 2 rRNA genes). The mitogenome of *Lepidostoma* found no gene rearrangement of gene order, which was the same as the ancestral gene order of *Drosophila yakuba* Burla, 1954 [45]. The gene cluster *trnR-trnN-trnS1-trnE-trnA-trnF* was found in the mitogenome of *Paraphlegopteryx.* The newly sequenced 14 mitogenomes of lepidostomatid ranged in length from 15,053 bp *Lepidostoma albardanum* Ulmer, 1906 to 16,495 bp *Paraphlegopteryx subcircularis* Schmid, 1965 (Table 1). Among them, the CRs of *Lepidostoma elongatum* Martynov, 1935 and *L. albardanum* could not complete the sequencing due to their complex structure and high AT content [46]. The incomplete CR did not affect subsequent phylogenetic analyses. The AT content varies from 76.59% in *Lepidostoma reductum* Martynov, 1915 to 80.40% in *P. subcircularis.* The AT content of whole mitogenomes were similar in other Plenitentoria species [26]. In the mitogenomes of lepidostomatid species, the AT skew was positive, while the GC skew was negative.

### 3.2. Protein-Coding Genes and Codon Usage

In the mitogenome, there are great variations in the length of each PCGs, which range from 165 bp (*ATP8*) to 1734 bp (*ND5*). There was no significant difference in the total length of 13 PCGs, ranging from 11,197 (*Lepidostoma aranos* Oláh, 2013) to 11,249 bp (*P. subcircularis*) (Table S3). For *ATP6*, *COX1*, *COX2 CYTB*, *ND1*, *ND2*, *ND4L*, and *ND5*, a fixed start codon is used in each gene, and more than two start codons are used in other genes (Figure S3). Except for *COX2*, the start codon of other PCGs is ATN mode. The termination codon for most PCGs, excluding *ND1*, is typically TAA or TAG, with TAA being the most frequently used. A single T is used as a termination of *ND1* in all species of *Lepidostoma*, while the common TAA was only used in *ND1* in *Paraphlegopteryx*, demonstrating inter-genus variability and intra-genus conservation. These incomplete stop codons are thought to have been filled with polyadenylation during mRNA maturation [24].

**Figure 3:**
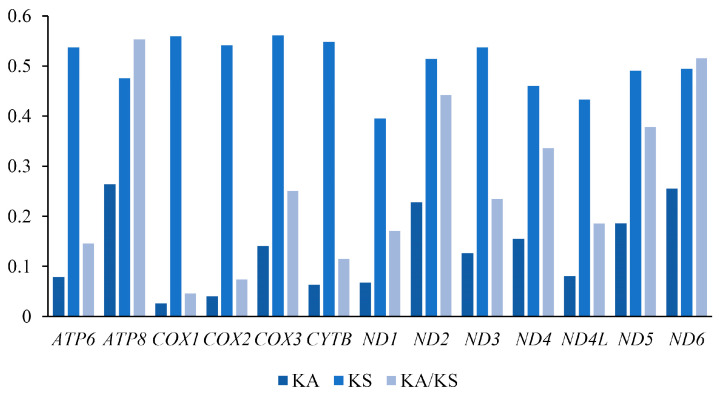
The evolution rate of PCGs of Lepidostomatidae. The abscissa represents the 13 PCGs, and the ordinate represents Ka/Ks values.

We used the Ka/Ks ratio to estimate the evolutionary rate of 13 PCGs (Figure 3). Our results showed that the Ka/Ks of 13 PCGs in the mitogenome of lepidostomatid species was less than 1, indicating that these genes were in a state of purified selection. The evolutionary rate of *ATP8* (0.55) is the highest, and the evolutionary rate of *COX1* (0.04) is the lowest, indicating that *ND6* has the lowest purification selection pressure, and *COX1* has the highest purification selection pressure, which has also been observed in other species of Integripalpia [2].

The analysis of nucleotide diversity based on 13 PCGs from 14 lepidostomatid species showed pi values ranging from 0.137 (*ND1*) to 0.241 (*ATP8*) (Table S4). The highest nucleotide diversity of *ATP8* gene indicates a large variation region, while *ND1* is a conserved gene in Lepidostomatidae. Therefore, *ND1* may be an effective molecular marker for species identification. The RSCU of these 14 species suggests a strong AT bias in the nucleotide composition of the mitogenome (Figures S4 and S5). The third codon position shows obvious A and T base dominance; TTA, ATT, TTT, ATA, and AAT are the most commonly used codons of these species. The results showed that Leu2, Phe, Ile, and Asn were the most commonly used amino acids.

### 3.3. Transfer RNAs and Ribosomal RNAs and Control Regions

The mitogenomes of each lepidostomatid species contain 22 tRNA genes, with lengths varying between 59 and 73 bp. Among the 22 tRNAs in the 14 mitogenomes, only the length of *trnV* is constant, and the length of the other tRNAs varies greatly or slightly. The GC-skew (0.171–0.101) and AT-skew (0.007–0.039) of tRNA genes were positive, and the AT content exceeded 82% (Table S3). The *l-rRNA* gene exhibits a size variation spanning from 1456 bp in *Lepidostoma propriopalpum* Hwang, 1957 to 1372 bp in *P. subcircularis*. In contrast, the *s-rRNA* gene shows a short variation, with the sequence observed ranging from 790 bp (*P. subcircularis*) to 804 bp (*L. reductum*). The nucleotide composition patterns in rRNA genes exhibit distinct differences compared to those in tRNA genes within the mitogenome. Analysis of rRNA genes revealed positive values for both GC-skew (ranging from 0.31 to 0.38) and AT-skew (ranging from 0.03 to 0.07), with the AT content surpassing 86% across all sequences. The lengths of the CR range from 159 bp to 1517 bp. The AT content of this region was the highest, ranging from 84.86% (*L. flavum*) to 91.61% (*L. hirtum*). Considering the length variation in tRNA and rRNA, the length variation in mitochondria is due to the length of the control region and gene spacer region, which is consistent with the results of other mitochondrial studies in metabiological animals.

### 3.4. Phylogenetic Analyses of Lepidostomatidae

**Figure 4:**
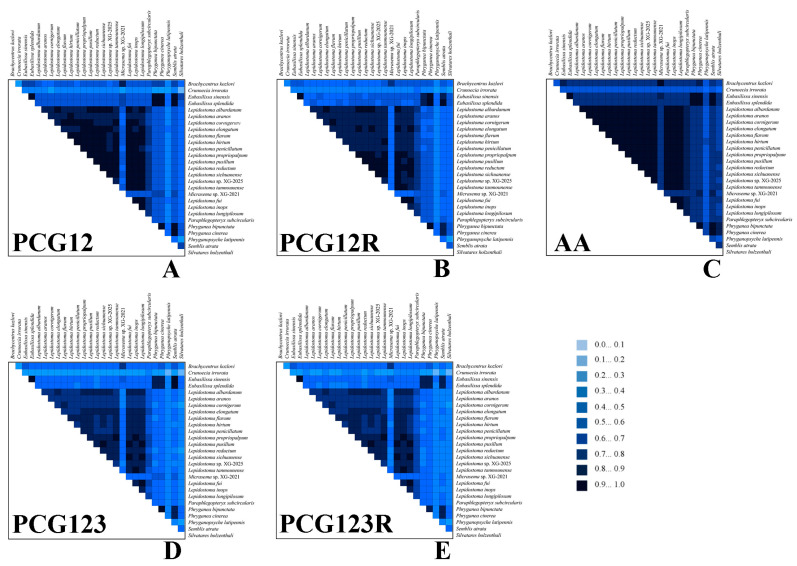
The evaluation of the heterogeneity among the five datasets. The degree of sequence similarity was visually represented through colored blocks, with scores in a spectrum from 0 (signaling major heterogeneity between datasets, represented by light blue) to +1 (signaling minor heterogeneity between datasets, depicted in dark blue). (**A**) PCG12; (**B**) PCG12R; (**C**) AA; (**D**) PCG123; (**E**) PCG123R.

In order to evaluate and understand the heterogeneity patterns of different datasets, we used AliGROOVE to perform heterogeneity analysis on the five generated datasets, (Figure 4) and the results showed that the amino acid dataset was the lowest in the five datasets, which was due to the amino acid sequence conservation caused by the use of synonymous codons [47]. Compared with the PCG123 dataset, the elimination of the third codon position can reduce the heterogeneity, which indicated that the evolution rate of the third codon position is faster. The nucleotide datasets with rRNA genes were less heterogeneous than those without rRNA genes. This is consistent with the results of mitogenome study in the Apataniidae by Ge et al. [2], suggesting that the addition of the rRNA gene can reduce heterogeneity during the reconstruction of the phylogenetic relationship using mitogenomes.

Notably, *Silvatares holzenthali* Rázuri-Gonzales, Ngera & Pauls, 2022 and *C. irrorata* showed significantly high sequence heterogeneity across all datasets. This phenomenon may be attributed to the accelerated rate of evolution of this species or limited phylogenetic resolution due to under-sampling of representatives of Pisuliidae and Theliopsychinae [2,48]. Saturation substitution analysis of the nucleotide dataset showed that the simple index of substitution saturation (ISS) was less than the critical ISS value (ISs.c), *p* < 0.05 (Figures S6 and S7). This suggests that these datasets were not saturated in terms of nucleotide substitution.

**Figure 5:**
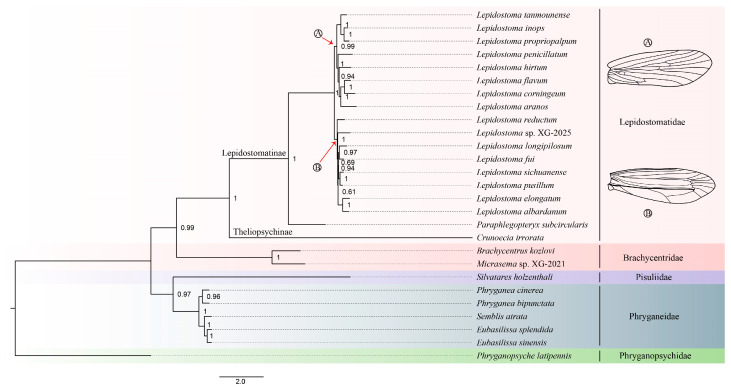
The BI tree based on the PCG123R dataset, using the CAT + GTR model. The numbers above nodes are Bayesian posterior probabilities. A: the *Lepidostoma hirtum* branch; B: the *Lepidostoma ferox* branch.

In this study, phylogenetic trees were reconstructed using ML and BI methods based on five datasets. The best partitions of five datasets for the ML analysis are listed in Table S5. The results of the phylogenetic tree show roughly similar topologies. In this study, the Lepidostomatidae was consistently recovered as monophyletic groups in all phylogenetic trees, which had high nodal support values (posterior probabilities PPs ≥ 95; bootstrap probabilities BPs ≥ 75). In the BI tree based on the PCG12R, *S. holzenthali* and two *Phryanea* species were recovered as an unresolved trichotomy (Figure S8). The phylogenetic tree based on the PCG123R dataset using the BI method supported topology (Phryganopsychidae + (Phryganeidae + Pisuliidae) + (Brachycentridae + Lepidostomatidae)) (Figure 5), while the ML trees based on the all datasets and BI tree based on PCG, PCG12, and AA datasets both supported topology (Phryganopsychidae + (((Phryganeidae + Pisuliidae) + Brachycentridae) + Lepidostomatidae)) (Figures S9–S16). When mitogenome is used in phylogenetic studies, different genes, coding position, and models may be selected to obtain different topologies. In the phylogenetic studies of insect groups, the use of two rRNA genes is more common than that of short tRNA genes with a lower evolutionary rate. Additionally, in phylogenetic studies of the orders Hemiptera and Hymenoptera, removal of the third codon position did not affect or even optimize the results, while in Diptera, this selection reduced the phylogenetic sign [49,50,51]. Comparing the studies of Paul and Ge et al. [52,53], we believe that the use of the BI model and the addition of the rRNA gene can improve the phylogenetic resolution in the study of trichopteran phylogeny (without an unresolved trichotomy, Figure 5). Therefore, we infer that Lepidostomatidae is a monophyletic family that is the sister group to Brachycentridae.

Within Lepidostomatidae, the monophyly of two subfamilies (Lepidostomatinae and Theliopsychinae) was strongly supported in all topologies. In *Lepidostoma*, four large species groups are distinguished by the structure of male forewings, with the most significant differences lying in the anal region. These varying forewing structures form a transitional series, which likely evolved in the sequence outlined below: *Lepidostoma vernale* branch, *Lepidostoma podagrum* branch, *Lepidostoma ferox* branch, and *Lepidostoma hirtum* branch. Weaver considered that the *L. vernale* and *L. hirtum* branches are each monophyletic, but suspected that the *L. podagrum* branch is not monophyletic [15]. The results of a phylogenetic analysis of most North American species using mtDNA by Myers and Sperling indicate that the *L. ferox* branch and *L. hirtum* branch are monophyletic [15,20].

Our phylogenetic results recovered monophyletic *L. ferox* and the *L. hirtum* branches, which is consistent with the results of Myers and Sperling [20]. The unnamed species *Lepidostoma* sp. XG-2025 might be classified under the *L. ferox* branch. It also indicates that the mitochondrial phylogenomics has provided effective data to help resolve some issues in the genus level phylogeny of Lepidostomatinae. Furthermore, we reconstructed the ML tree using Theliopsychinae as the outer group and Lepidostomatinae as the inner group (Figures S17–S21). The interspecific phylogenetic relationships in these phylogenetic trees are roughly the same. In the *L. hirtum* branch, *Lepidostoma inops* species group (*L. tanmounense* + (*L. inops* + *L. propriopalpum*)) was recovered a monophyletic group, which supports the classification of the species group. In the *L. ferox* branch, Species with similar geographical distributions (*L. albardanum* and *L. elongatum*; *L. sichuanense and L. pusillum*) have a closer genetic relationship. In our initial phylogenetic study, the *L. vernale* and *L. podagrum* branches distributed in North America were not included. Although the *L. ferox* and *L. hirtum* branches were restored to sister groups in this study, this might not reflect the true phylogenetic relationship among each branch. Therefore, the current molecular studies on Lepidostomatidae are limited. In this study, the mitogenome of Lepidostomatidae was added, which provides scientific basis for female and larval identification and evolutionary trend of Lepidostomatidae. However, the sample and molecular data in this study are limited. Future studies should include more taxa samples (*Paraphlegopteryx*, *Zephyropsyche*, *Theliopsyche* and *Lepidostoma* species) and more molecular data (Universal Single-Copy Orthologs or Ultra-Conserved Elements) to facilitate the understanding of phylogenetic relationships in Lepidostomatidae.

## 4. Conclusions

In this study, we sequenced and compared the mitogenomes of 14 lepidostomatid species and reported the mitogenome structure of *Paraphlegopteryx* for the first time. The results showed that the mitogenome of Lepidostomatidae was similar to that of most other insects. The phylogenetic relationship of Lepidostomatidae was reconstructed using ML and BI methods based on mitogenomes. Phylogenetic results showed that the family Lepidostomatidae was consistently recovered as monophyletic groups, and the taxonomic position of the two subfamilies was strongly supported. In addition, we recovered monophyletic *L. ferox* and *L. hirtum* branches. This preliminary result suggests that future studies need to clarify phylogenetic relationships within Lepidostomatidae by increasing the number of taxon samples and integrate more molecular data.

## Figures and Tables

**Table 1:** Nucleotide composition of 14 species of Lepidostomatinae.

Species	Length (bp)	A (%)	T (%)	C (%)	G (%)	A + T (%)	GC-Skew	AT-Skew
*Lepidostoma albardanum*	15,053	38.82	39.40	14.34	7.43	78.22	−0.317	−0.007
*Lepidostoma elongatum*	15,234	39.13	40.47	13.13	7.26	79.60	−0.288	−0.017
*Lepidostoma aranos*	15,870	38.49	39.19	15.00	7.30	77.67	−0.345	−0.009
*Lepidostoma cornigerum*	15,325	38.04	39.11	15.37	7.48	77.15	−0.346	−0.014
*Lepidostoma flavum*	15,816	38.12	40.05	14.53	7.23	78.17	−0.335	−0.025
*Lepidostoma hirtum*	15,663	38.75	39.45	14.45	7.35	78.20	−0.325	−0.009
*Lepidostoma penicillatum*	15,875	39.04	40.34	13.44	7.15	79.38	−0.305	−0.016
*Lepidostoma propriopalpum*	15,158	39.12	40.32	13.28	7.27	79.44	−0.293	−0.015
*Lepidostoma pusillum*	16,036	38.83	39.92	13.81	7.39	78.74	−0.303	−0.014
*Lepidostoma reductum*	15,077	38.56	38.03	15.24	8.17	76.59	−0.302	0.007
*Lepidostoma sichuanense*	16,165	38.47	39.65	14.24	7.52	78.11	−0.309	−0.015
*Lepidostoma* sp. XG-2025	15,115	38.86	39.05	14.49	7.61	77.90	−0.311	−0.002
*Lepidostoma tanmounense*	15,065	38.58	40.07	13.88	7.47	78.65	−0.300	−0.019
*Paraphlegopteryx subcircularis*	16,495	38.87	41.53	12.57	6.80	80.40	−0.298	−0.033

## Data Availability

The data of this study are available under the NCBI accession numbers (PV364373, PV364374 and PV366289–PV366300).

## Supplementary Materials

The following supporting information can be downloaded at https://www.mdpi.com/article/10.3390/insects16050536/s1. Table S1. The taxonomic information and sampling metadata of the sequenced samples. Table S2. Detailed taxonomic resources used in phylogeny analysis. Table S3. Nucleotide composition of 14 lepidostomatid mitogenomes. Table S4. Nucleotide diversity (Pi) values of 14 lepidostomatid species mitogenomes. Table S5. The best partitions of five datasets for the ML analysis. Figures S1 and S2. The mitogenome map of the 12 lepidostomatid species mitogenome. Figure S3. Strat codons and termination codons of PCGs among 14 lepidostomatid species mitogenomes. Figures S4 and S5. The relative synonymous codon usage of the PCGs of 14 lepidostomatid species. Figures S6 and S7. Substitution saturation plots of PCG123 and PCG12 datasets. Figures S8–S11. The BI trees based on the PCG12R, PCG12, PCG, and AA dataset, using the CAT + GTR model. Figures S12–S16. The ML trees based on the PCG123R, PCG12R, PCG12, PCG, and AA dataset, using the Partitioning model. Figures S17–S21. The ML tree based on the AA, PCG, PCG123R, PCG12, PCG12R dataset of Lepidostomatidae, using the Partitioning model. The numbers above nodes are bootstrap probabilities.
