# Supplementary material for: Phylogenetic Analysis of the Family Lepidostomatidae (Trichoptera: Integripalpia) Using Whole Mitochondrial Genomes
Source: Insects. 2025 May 19;16(5):536. doi: 10.3390/insects16050536 (PMC12111908; doi:10.3390/insects16050536)
Supplement: Supplementary file 1 [file insects-16-00536-s001.zip › insects-3576521-supplementary.pdf]

Table S1. Information on the collection of the newly sequenced samples.

Family	Species	Sample ID	Life Stage	Sampling Metadata
Lepidostomatidae	*Lepidostoma albardanum*	LNBX-003	Adult male	Caohezhang Town, Benxi City, Liaoning Province, China<br>41°7'15.384" N, 124°14'17.844" E, 26 Jul 2023, leg, XY. Ge
Lepidostomatidae	*Lepidostoma aranos*	JFL-021	Adult male	Jianfengling, Ledong County, Hainan Province, China<br>18°44'36.672" N, 108°51'13.428" E, 20 Dec 2024, leg, XY. Ge
Lepidostomatidae	*Lepidostoma cornigerum*	FJ-D4-19	Adult male	201 Township Road, Nanping City, Fujian Province, China<br>27°50'14.446" N, 117°58'11.561" E, 22 Jul 2022, Malaise trap
Lepidostomatidae	*Lepidostoma elongatum*	LNDB-202	Adult male	Da Binggou, Benxi City, Liaoning Province, China<br>41°6'22.144" N, 123°55'50.683" E, 30 Jul 2023, leg, XY. Ge
Lepidostomatidae	*Lepidostoma flavum*	SDYT-005	Adult male	Kunyu Mountain, Yantai City, Shandong Province, China<br>37°16'51.888" N, 121°43'33.348" E, 28 May 2024, leg, JY. Wang
Lepidostomatidae	*Lepidostoma hirtum*	HJL-012	Adult male	Huma River, Huma County, Heilongjiang Province, China<br>51°39'52.618" N, 126°36'27.770" E, 14 Jul 2024, leg, DW Gong
Lepidostomatidae	*Lepidostoma penicillatum*	XJXY-003	Adult male	Kurdning Nature Reserve, Xinyuan County, Xinjiang, China<br>43°9'6.120" N, 82°52'22.080" E, 27 Jul 2024, leg, XY. Ge
Lepidostomatidae	*Lepidostoma propriopalpum*	ZJLS-021	Adult male	Xijing Village, Lishui City, Zhejiang Province, China<br>28°10'31.248" N, 118°49'2.453" E, 1–10 Jul 2023, Malaise trap
Lepidostomatidae	*Lepidostoma pusillum*	SCHL-046	Adult male	Huanglong Wetland, Songpan County, Sichuan Province, China<br>32°44'43.338" N, 103°45'20.762" E, 23 Jul 2024, leg, XY. Ge, W. C
Lepidostomatidae	*Lepidostoma reductum*	XJXY-002	Adult male	Kurdning Nature Reserve, Xinyuan County, Xinjiang, China<br>43°9'6.120" N, 82°52'22.080" E, 27 Jul 2024, leg, XY. Ge
Lepidostomatidae	*Lepidostoma sichuanense*	5117	Adult male	Huanglong Wetland, Songpan County, Sichuan Province, China<br>32°47'41.280" N, 103°54'27.000" E, 1 Aug 2024, leg, XY. Ge, W. C
Lepidostomatidae	*Lepidostoma* sp. XG-2025	YGL-021	Adult male	Yingge Ridge, Qiongzhong County, Hainan Province, China<br>19°1'33.204" N, 109°34'11.690" E, 21 Dec 2024, leg, XY. Ge

Lepidostomatidae	*Lepidostoma tanmounense*	WYS-127	Adult male	Mount Wuyi, Nanping City, Fujian Province, China 27°41'24.000" N, 117°39'0.000" E, 1–15 Jul 2024, Malaise trap
Lepidostomatidae	*Paraphlegopteryx subcircularis*	wysPSWSA	Adult male	Mount Wuyi, Nanping City, Fujian Province, China 27°41'24.000" N, 117°39'0.000" E, 1–15 Jul 2024, Malaise trap

Table S2. Detailed taxonomic resources used in present study.

	Family	Genus	Species	GenBank access
ingroup	Lepidostomatidae	*Crunoecia*	*Crunoecia irrorata* (Curtis, 1834)	MT874486
		Lepidostoma	*Lepidostoma albardanum* (Ulmer, 1906)	PV364373
			Lepidostoma aranos Oláh, 2013	PV366289
			Lepidostoma cornigerum (Ulmer, 1907)	PV366290
			Lepidostoma elongatum (Martynov, 1935)	PV364374
			Lepidostoma flavum (Ulmer, 1926)	PV366291
			Lepidostoma fui (Hwang, 1957)	OL678026
			Lepidostoma hirtum (Fabricius, 1775)	PV366292
			Lepidostoma inops (Ulmer, 1926)	OL678027
			Lepidostoma longipilosum (Schmid, 1965)	OL677992
			Lepidostoma penicillatum (McLachlan, 1875)	PV366293
			Lepidostoma propriopalpum (Hwang, 1957)	PV366294
			Lepidostoma pusillum (Martynov, 1931)	PV366295
			Lepidostoma reductum (Martynov, 1915)	PV366296
			Lepidostoma sichuanense (Yang & Weaver, 2002)	PV366297
			Lepidostoma tanmounense (Hsu & Chen, 1996)	PV366299
			Lepidostoma sp. XG-2025	PV366298
		Paraphlegopteryx	*Paraphlegopteryx subcircularis* Schmid, 1965)	PV366300
outgroup	Brachycentridae		*Brachycentrus kozlovi* Martynov, 1909	OL678005

	Micrasema sp. XG-2021	OL677994
Phryganeidae	*Eubasilissa sinensis* Schmid, 1959	OL678014
	Eubasilissa splendida Yang & Yang, 2006	OL678015
	Phryganea bipunctata Retzius, 1783	MT410848
	Phryganea cinerea Walker, 1852	MG980616
	Semblis atrata (Gmelin, 1789)	MZ514855
Phryganopsychidae	*Phryganopsyche latipennis* (Banks, 1906)	KX385012
Pisuliidae	*Silvatares holzenthali* (Rázuri-Gonzales, Ngera & Pauls, 2022)	OP921089

Table S3. Nucleotide composition of 14 mitogenomes.

Species	Regions	length (bp)	A (%)	T (%)	C (%)	G (%)	A+T (%)	G+C (%)	AT -Skew	GC -Skew
Lepidostoma albardanum	Whole genome	15,053	38.82	39.40	14.34	7.43	78.22	21.78	-0.007	-0.317
	PCGs	11,200	33.18	43.61	12.90	10.31	76.79	23.21	-0.136	-0.112
	Site 1	3,734	37.92	36.27	12.22	13.60	74.18	25.82	0.022	0.054
	Site 2	3,733	22.17	49.91	15.65	12.27	72.08	27.92	-0.385	-0.121
	Site 12	7,467	30.05	43.09	13.93	12.93	73.13	26.87	-0.178	-0.037
	Site 3	3,733	39.45	44.65	10.84	5.05	84.10	15.90	-0.062	-0.364
	tRNA	1,457	42.42	40.97	7.28	9.33	83.39	16.61	0.017	0.124
	l-rRNA	1,374	46.58	40.17	4.22	9.02	86.75	13.25	0.074	0.363
	s-rRNA	791	45.76	42.23	4.17	7.84	87.99	12.01	0.040	0.305
	CR	159	35.22	51.57	10.69	2.52	86.79	13.21	-0.188	-0.619
Lepidostoma aranos	Whole genome	15,870	38.49	39.19	15.00	7.30	77.67	22.31	-0.009	-0.345
	PCGs	11,197	32.44	42.80	14.06	10.71	75.24	24.76	-0.138	-0.135
	Site 1	3,733	37.41	35.40	13.10	14.09	72.82	27.18	0.028	0.036
	Site 2	3,732	21.75	49.87	16.40	11.98	71.62	28.38	-0.393	-0.156
	Site 12	7,465	29.58	42.64	14.75	13.03	72.22	27.78	-0.181	-0.062
	Site 3	3,732	38.15	43.12	12.67	6.06	81.28	18.72	-0.061	-0.353
	tRNA	1,458	43.62	40.33	7.00	9.05	83.95	16.05	0.039	0.128
	l-rRNA	1,378	44.12	41.29	4.14	10.45	85.41	14.59	0.033	0.433
	s-rRNA	792	45.20	42.42	4.29	8.08	87.63	12.37	0.032	0.306
	CR	1,010	38.02	52.97	6.73	1.98	90.99	8.71	-0.164	-0.546
Lepidostoma cornigerum	Whole genome	15,325	38.04	39.11	15.37	7.48	77.15	22.85	-0.014	-0.346
	PCGs	11,206	32.06	43.44	13.94	10.56	75.50	24.50	-0.151	-0.138
	Site 1	3,736	37.41	36.16	12.83	13.60	73.57	26.43	0.017	0.029
	Site 2	3,735	21.72	50.12	16.14	12.03	71.83	28.17	-0.395	-0.146
	Site 12	7,471	29.56	43.14	14.49	12.81	72.70	27.30	-0.187	-0.061
	Site 3	3,735	37.06	44.04	12.85	6.05	81.10	18.90	-0.086	-0.359
	tRNA	1,453	42.26	40.67	7.36	9.70	82.93	17.07	0.019	0.137
	l-rRNA	1,381	45.47	41.49	4.56	8.47	86.97	13.03	0.046	0.300
	s-rRNA	799	47.18	40.80	3.88	8.14	87.98	12.02	0.073	0.354
	CR	490	35.10	51.63	10.41	2.86	86.73	13.27	-0.191	-0.569
Lepidostoma elongatum	Whole genome	15,234	39.13	40.47	13.13	7.26	79.60	20.39	-0.017	-0.288
	PCGs	11,206	33.29	45.08	11.75	9.87	78.37	21.63	-0.150	-0.087
	Site 1	3,736	37.44	37.36	11.74	13.45	74.81	25.19	0.001	0.068
	Site 2	3,735	21.92	50.31	15.65	12.12	72.23	27.77	-0.393	-0.127
	Site 12	7,471	29.68	43.83	13.70	12.79	73.52	26.48	-0.193	-0.034
	Site 3	3,735	40.51	47.57	7.87	4.05	88.09	11.91	-0.080	-0.320
	tRNA	1,461	43.74	40.59	7.05	8.62	84.33	15.67	0.037	0.100
	l-rRNA	1,385	46.43	40.43	4.48	8.66	86.86	13.14	0.069	0.319
	s-rRNA	795	46.92	40.88	3.90	8.30	87.80	12.20	0.069	0.361
	CR	345	35.65	52.17	8.41	3.19	87.83	11.59	-0.188	-0.450

	Whole genome	15,816	38.12	40.05	14.53	7.23	78.17	21.76	-0.025	-0.335
	PCGs	11,203	32.47	43.92	13.11	10.50	76.39	23.61	-0.150	-0.111
	Site 1	3,735	37.21	35.94	12.85	14.00	73.15	26.85	0.017	0.043
	Site 2	3,734	21.77	50.14	15.87	12.22	71.91	28.09	-0.394	-0.130
Lepidostoma	Site 12	7,469	29.49	43.04	14.36	13.11	72.53	27.47	-0.187	-0.046
flavum	Site 3	3,734	38.42	45.68	10.61	5.28	84.11	15.89	-0.086	-0.335
	tRNA	1,446	42.95	40.25	7.54	9.27	83.20	16.80	0.032	0.103
	l-rRNA	1,386	45.82	41.27	4.11	8.80	87.09	12.91	0.052	0.363
	s-rRNA	803	47.45	40.22	3.99	8.34	87.67	12.33	0.082	0.354
	CR	984	35.67	49.19	12.09	1.93	84.86	14.02	-0.159	-0.725
	Whole genome	15,663	38.75	39.45	14.45	7.35	78.20	21.80	-0.009	-0.325
	PCGs	11,200	32.59	43.91	13.04	10.46	76.50	23.50	-0.148	-0.110
	Site 1	3,734	37.57	36.90	11.89	13.64	74.47	25.53	0.009	0.069
	Site 2	3,733	22.26	50.20	15.62	11.92	72.46	27.54	-0.386	-0.135
Lepidostoma	Site 12	7,467	29.92	43.55	13.75	12.78	73.47	26.53	-0.186	-0.037
hirtum	Site 3	3,733	37.94	44.62	11.62	5.82	82.56	17.44	-0.081	-0.333
	tRNA	1,451	42.94	40.45	7.03	9.58	83.39	16.61	0.030	0.154
	l-rRNA	1,419	45.10	41.37	3.95	9.58	86.47	13.53	0.043	0.417
	s-rRNA	801	44.44	42.95	4.24	8.36	87.39	12.61	0.017	0.327
	CR	775	41.55	50.06	7.10	1.29	91.61	8.39	-0.093	-0.692
	Whole genome	15,875	39.04	40.34	13.44	7.15	79.38	20.59	-0.016	-0.305
	PCGs	11,204	33.41	44.44	12.28	9.87	77.85	22.15	-0.142	-0.109
	Site 1	3,732	38.24	36.17	12.28	13.31	74.41	25.59	0.028	0.040
	Site 2	3,730	22.06	50.00	15.89	12.05	72.06	27.94	-0.388	-0.137
Lepidostoma	Site 12	7,462	30.15	43.08	14.08	12.68	73.23	26.77	-0.177	-0.052
penicillatum	Site 3	3,730	39.90	47.14	8.69	4.27	87.04	12.96	-0.083	-0.341
	tRNA	1,448	43.37	40.47	6.77	9.39	83.84	16.16	0.035	0.162
	l-rRNA	1,390	46.62	40.36	4.10	8.92	86.98	13.02	0.072	0.370
	s-rRNA	796	45.10	42.34	4.40	8.17	87.44	12.56	0.032	0.300
	CR	1,002	38.22	47.90	10.18	3.09	86.13	13.27	-0.112	-0.534
	Whole genome	15,158	39.12	40.32	13.28	7.27	79.44	20.55	-0.015	-0.293
	PCGs	11,206	33.68	44.61	11.95	9.76	78.28	21.71	-0.140	-0.101
	Site 1	3,736	37.78	36.76	12.00	13.44	74.54	25.44	0.014	0.057
	Site 2	3,735	22.19	50.30	15.63	11.87	72.49	27.49	-0.388	-0.137
Lepidostoma	Site 12	7,471	29.99	43.53	13.81	12.65	73.52	26.47	-0.184	-0.044
propriopalpum	Site 3	3,735	41.06	46.77	8.21	3.96	87.82	12.18	-0.065	-0.349
	tRNA	1,457	43.38	40.84	6.73	9.06	84.21	15.79	0.030	0.148
	l-rRNA	1,456	46.29	40.93	4.19	8.59	87.23	12.77	0.061	0.344
	s-rRNA	798	46.12	41.98	4.14	7.77	88.10	11.90	0.047	0.305
	CR	208	37.02	52.40	7.21	3.37	89.42	10.58	-0.172	-0.364
	Whole genome	16,036	38.83	39.92	13.81	7.39	78.74	21.20	-0.014	-0.303
Lepidostoma	PCGs	11,203	33.04	43.87	12.34	10.76	76.91	23.09	-0.141	-0.068
pusillum	Site 1	3,735	37.28	36.30	12.28	14.14	73.58	26.42	0.013	0.070
	Site 2	3,734	22.08	49.92	15.72	12.28	72.00	28.00	-0.387	-0.123

	Site 12	7,469	29.68	43.11	14.00	13.21	72.79	27.21	-0.185	-0.029
	Site 3	3,734	39.76	45.38	9.01	5.85	85.15	14.85	-0.066	-0.213
	tRNA	1,463	42.58	40.94	6.97	9.50	83.53	16.47	0.020	0.154
	l-rRNA	1,413	47.06	40.62	4.03	8.28	87.69	12.31	0.073	0.345
	s-rRNA	795	46.16	42.01	4.28	7.55	88.18	11.82	0.047	0.277
	CR	1,097	36.46	48.86	10.57	3.28	85.32	13.86	-0.145	-0.526
Lepidostoma reductum	Whole genome	15,077	38.56	38.03	15.24	8.17	76.59	23.41	0.007	-0.302
	PCGs	11,199	32.17	42.64	13.70	11.49	74.81	25.19	-0.140	-0.088
	Site 1	3,735	36.88	35.81	12.78	14.53	72.69	27.31	0.015	0.064
	Site 2	3,734	22.02	50.04	15.36	12.58	72.06	27.94	-0.389	-0.099
	Site 12	7,469	29.45	42.93	14.07	13.55	72.38	27.62	-0.186	-0.019
	Site 3	3,734	37.63	42.07	12.96	7.34	79.70	20.30	-0.056	-0.277
	tRNA	1,454	42.50	40.99	7.22	9.28	83.49	16.51	0.018	0.125
	l-rRNA	1,412	47.45	39.87	4.18	8.50	87.32	12.68	0.087	0.341
	s-rRNA	804	45.40	43.03	3.98	7.59	88.43	11.57	0.027	0.312
	CR	161	40.37	45.34	11.80	2.48	85.71	14.29	-0.058	-0.652
Lepidostoma sichuanense	Whole genome	16,165	38.47	39.65	14.24	7.52	78.11	21.76	-0.015	-0.309
	PCGs	11,203	33.13	42.84	13.38	10.66	75.96	24.04	-0.128	-0.113
	Site 1	3,735	37.54	35.40	12.96	14.10	72.94	27.06	0.029	0.042
	Site 2	3,734	22.39	50.27	15.14	12.20	72.66	27.34	-0.384	-0.108
	Site 12	7,469	29.97	42.84	14.05	13.14	72.81	27.19	-0.177	-0.033
	Site 3	3,734	39.45	42.83	12.04	5.67	82.28	17.72	-0.041	-0.360
	tRNA	1,461	41.96	40.38	7.32	10.34	82.34	17.66	0.019	0.171
	l-rRNA	1,404	46.58	40.31	4.13	8.97	86.89	13.11	0.072	0.370
	s-rRNA	796	45.48	41.96	4.40	8.17	87.44	12.56	0.040	0.300
	CR	1,250	38.40	49.60	8.16	2.24	88.00	10.40	-0.127	-0.569
Lepidostoma sp. XG-2025	Whole genome	15,115	38.86	39.05	14.49	7.61	77.90	22.10	-0.002	-0.311
	PCGs	11,197	33.22	43.27	13.29	10.22	76.49	23.51	-0.131	-0.130
	Site 1	3,733	38.13	35.27	12.82	13.78	73.40	26.60	0.039	0.036
	Site 2	3,732	22.22	50.36	15.23	12.19	72.57	27.43	-0.388	-0.111
	Site 12	7,465	30.17	42.81	14.02	12.99	72.99	27.01	-0.173	-0.038
	Site 3	3,732	39.31	44.19	11.81	4.69	83.50	16.50	-0.059	-0.432
	tRNA	1,463	42.45	41.42	7.04	9.09	83.87	16.13	0.012	0.127
	l-rRNA	1,396	45.85	40.62	4.01	9.53	86.46	13.54	0.060	0.407
	s-rRNA	797	45.17	42.79	3.89	8.16	87.95	12.05	0.027	0.354
	CR	264	37.50	48.48	10.23	3.79	85.98	14.02	-0.128	-0.460
Lepidostoma tanmounense	Whole genome	15,065	38.58	40.07	13.88	7.47	78.65	21.35	-0.019	-0.300
	PCGs	11,697	32.52	44.56	12.45	10.48	77.07	22.93	-0.156	-0.086
	Site 1	3,735	37.41	36.84	11.78	13.96	74.25	25.75	0.008	0.085
	Site 2	3,734	21.58	50.46	16.12	11.84	72.04	27.96	-0.401	-0.153
	Site 12	7,469	29.49	43.65	13.95	12.90	73.15	26.85	-0.194	-0.039
	Site 3	3,733	39.06	47.08	9.36	4.51	86.13	13.87	-0.093	-0.350
	tRNA	1,459	43.39	40.44	6.92	9.25	83.82	16.18	0.035	0.144
	l-rRNA	1,411	46.70	40.33	4.11	8.86	87.03	12.97	0.073	0.366

	s-rRNA	791	46.65	41.85	4.05	7.46	88.50	11.50	0.054	0.297
	CR	159	36.48	50.94	10.69	1.89	87.42	12.58	-0.165	-0.700
Paraphlegopteryx subcircularis	Whole genome	16,495	38.87	41.53	12.57	6.80	80.40	19.37	-0.033	-0.298
	PCGs	11,249	33.73	44.35	11.81	10.10	78.08	21.92	-0.136	-0.078
	Site 1	3,748	38.32	36.73	11.22	13.73	75.05	24.95	0.021	0.100
	Site 2	3,747	21.99	50.01	15.94	12.05	72.01	27.99	-0.389	-0.139
	Site 12	7,495	30.16	43.37	13.58	12.89	73.53	26.47	-0.180	-0.026
	Site 3	3,747	40.81	46.34	8.31	4.54	87.14	12.86	-0.063	-0.293
	tRNA	1,479	42.66	42.06	6.42	8.86	84.72	15.28	0.007	0.159
	l-rRNA	1,372	46.50	39.80	4.15	9.55	86.30	13.70	0.078	0.394
	s-rRNA	790	46.84	41.27	4.05	7.85	88.10	11.90	0.063	0.319
	CR	1,517	35.40	54.71	6.00	1.38	90.11	7.38	-0.214	-0.625

Table S4. Nucleotide diversity (Pi) values of 14 lepidostomatid species of PCGs.

Gene	Nucleotide diversity (Pi)
ATP6	0.181
ATP8	0.241
COX1	0.147
COX2	0.142
COX3	0.170
CYTB	0.167
ND1	0.137
ND2	0.205
ND3	0.206
ND4	0.158
ND4L	0.149
ND5	0.196
ND6	0.225

Table S5. Final gene partitions for the Maximum Likelihood Phylogenetic analysis

Matrix	Partition names	Best model
PCGAA	*ATP6, COX1, COX2, COX3, CYTB, ND3*	mtART+F+R4
	ATP8, ND2, ND6	mtMAM+F+R4
	ND1, ND4, ND4L, ND5	mtMet+F+R5
PCG123	*ATP6, ND3*	GTR+F+I+G4
	ATP8, ND2, ND6, COX1, COX2, COX3, CYTB	GTR+F+R4
	ND1, ND4L, ND5	GTR+F+R5
	ND4	GTR+F+R3
PCG123R	*ATP6, ND3*	GTR+F+I+G4
	ATP8, ND2, ND6, COX1, COX2, COX3, CYTB	GTR+F+R4
	ND1, ND4L, ND5	GTR+F+R5
	ND4, l- rRNA, s-rRNA	GTR+F+R3
PCG12	*ATP6, COX2, COX3, CYTB*	GTR+F+I+G4
	ATP8, ND2, ND3, ND6	GTR+F+R4

	COX1	TIM+F+I+G4
	ND1, ND5	TIM+F+R3
	ND4, ND4L	K3Pu+F+R2
PCG12R	*ATP6, COX2, COX3, CYTB*	GTR+F+I+G4
	ATP8, ND2, ND3, ND6	GTR+F+R4
	COX1	TIM+F+I+G4
	ND1, ND5	TIM+F+R3
	ND4, ND4L	K3Pu+F+R2
	l-rRNA, s-rRNA	GTR+F+R3

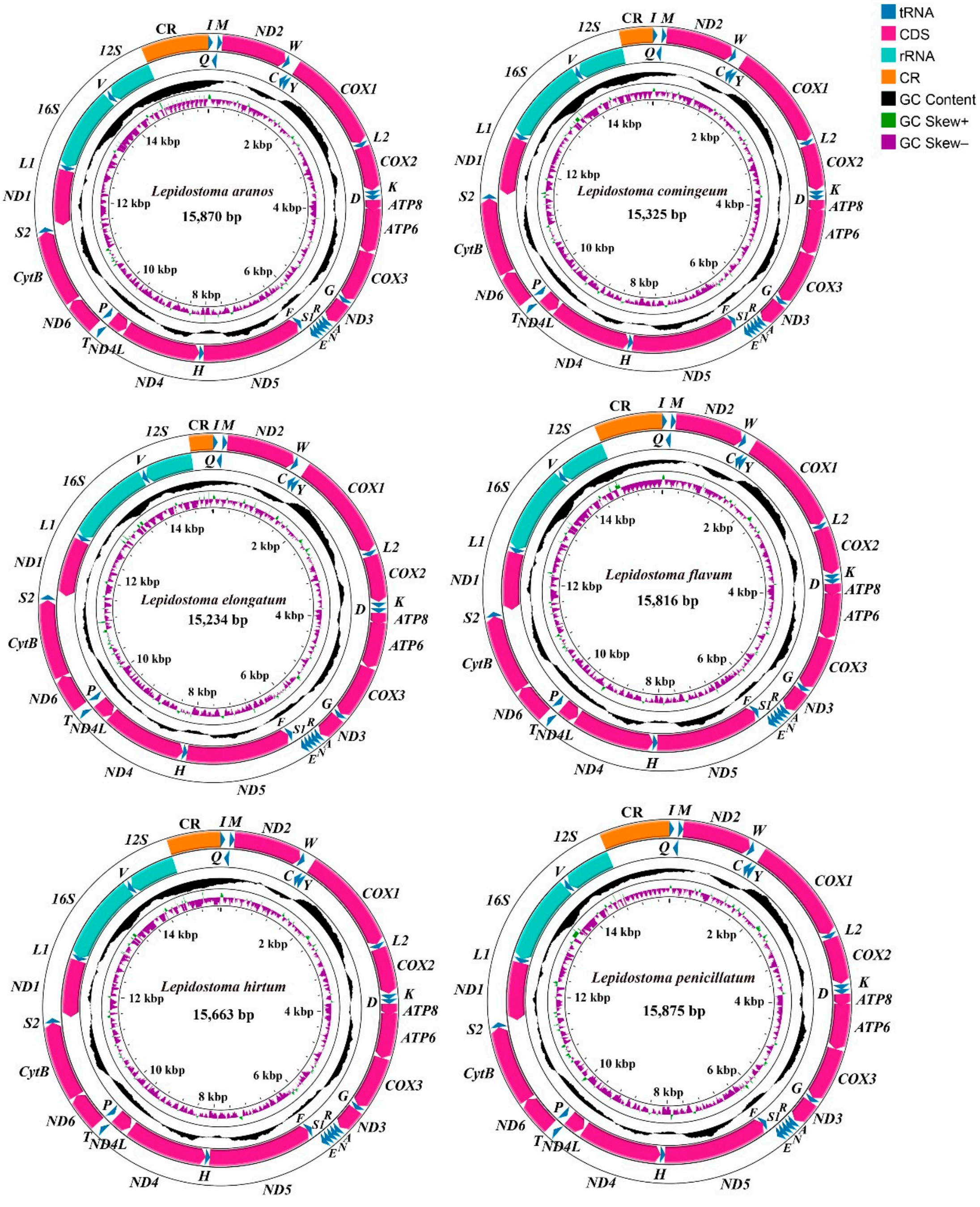

Figure S1. The mitogenome map of the six lepidostomatid species mitogenome. The arrow served as a guide, pointing to the orientation of gene transcription. We used standardized abbreviations to denote PCGs and rRNAs, while single-letter abbreviations were chosen for tRNAs. The second circle highlighted the GC content of the entire mitogenome, whereas the third circle revealed the GC-skew.

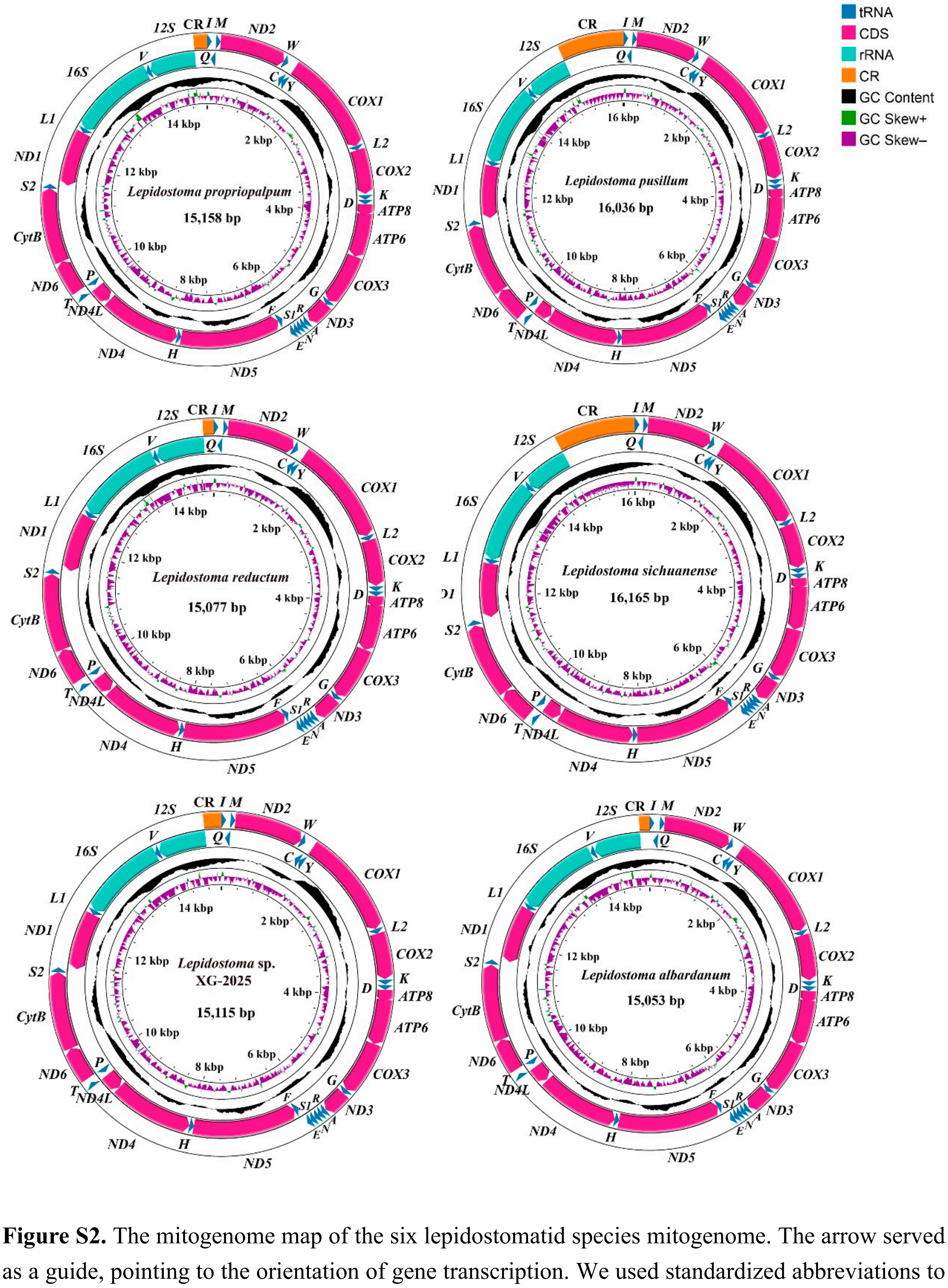

Figure S2. The mitogenome map of the six lepidostomatid species mitogenome. The arrow served as a guide, pointing to the orientation of gene transcription. We used standardized abbreviations to denote PCGs and rRNAs, while single-letter abbreviations were chosen for tRNAs. The second circle highlighted the GC content of the entire mitogenome, whereas the third circle revealed the GC-skew.

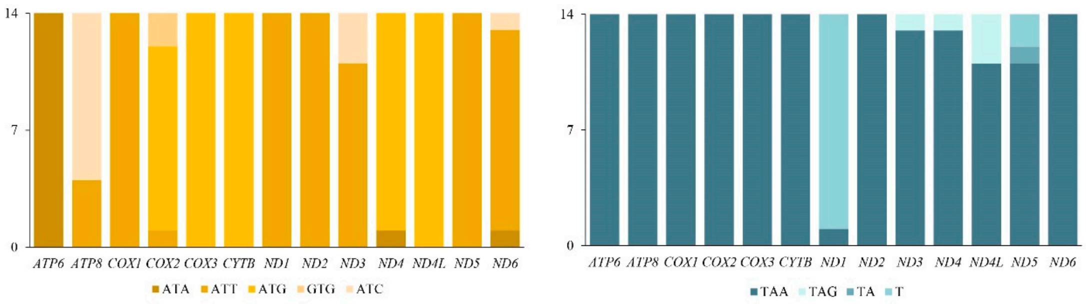

Figure S3. Strat codons and termination codons of PCGs among 14 lepidostomatid species mitogenomes.

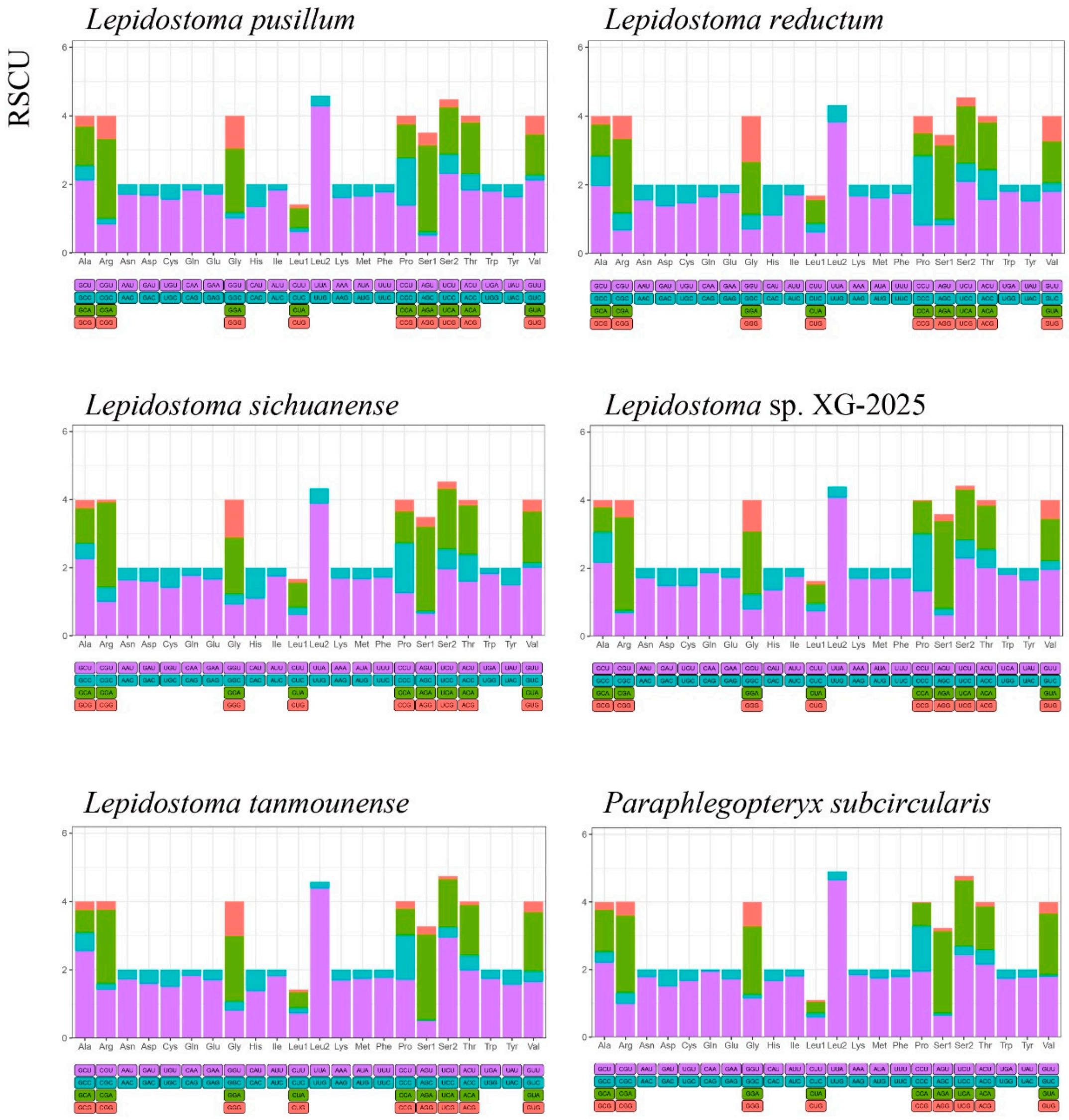

Figure S4. The relative synonymous codon usage of the PCGs of six lepidostomatid species. The X-axis shows 20 amino acids, and the Y-axis shows the number of times a certain synonymous codon is used/the average number of times that all codons coding the amino acid are used

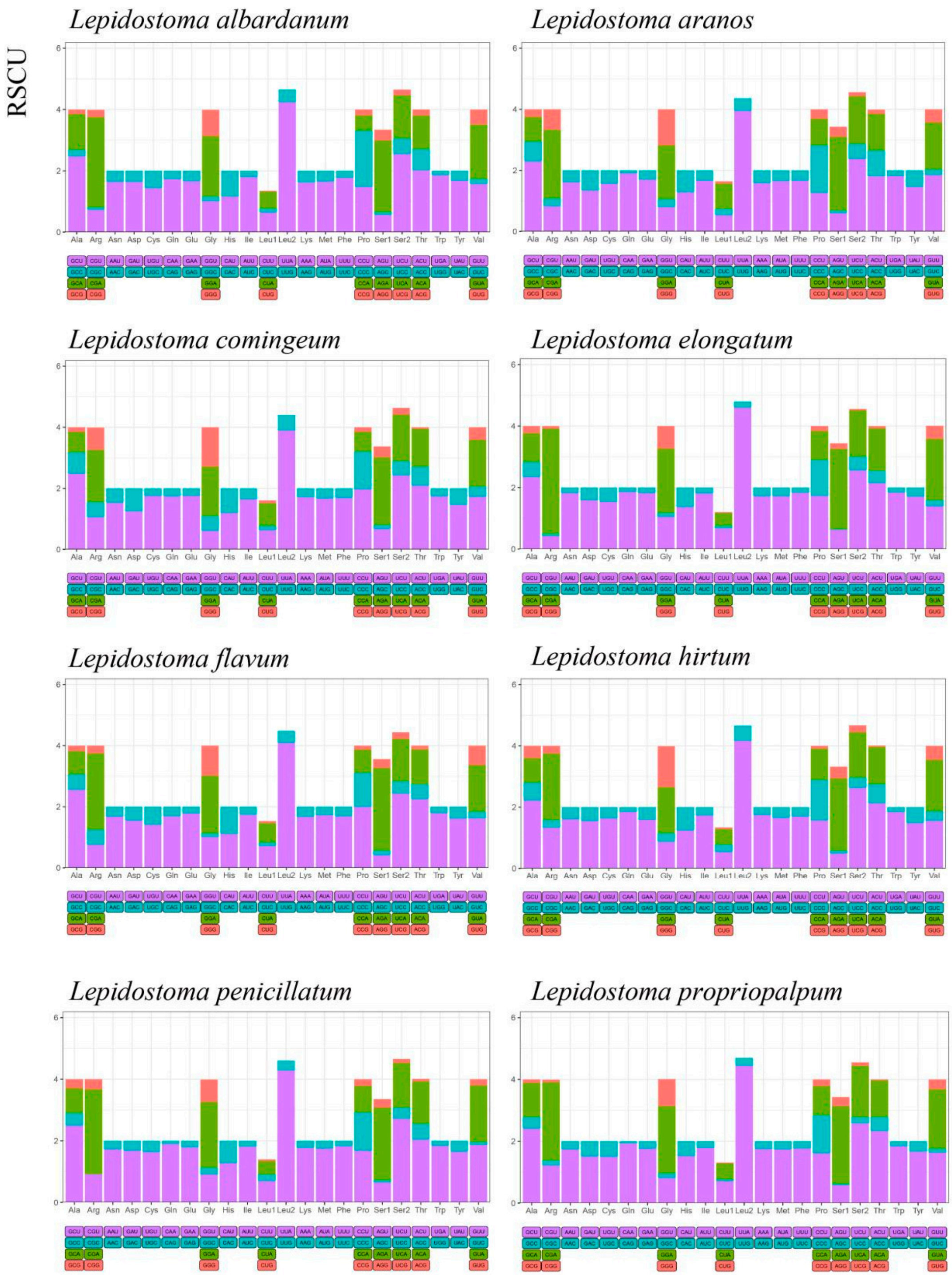

Figure S5. The relative synonymous codon usage of the PCGs of eight lepidostomatid species. The X-axis shows 20 amino acids, and the Y-axis shows the number of times a certain synonymous codon is used/the average number of times that all codons coding the amino acid are used

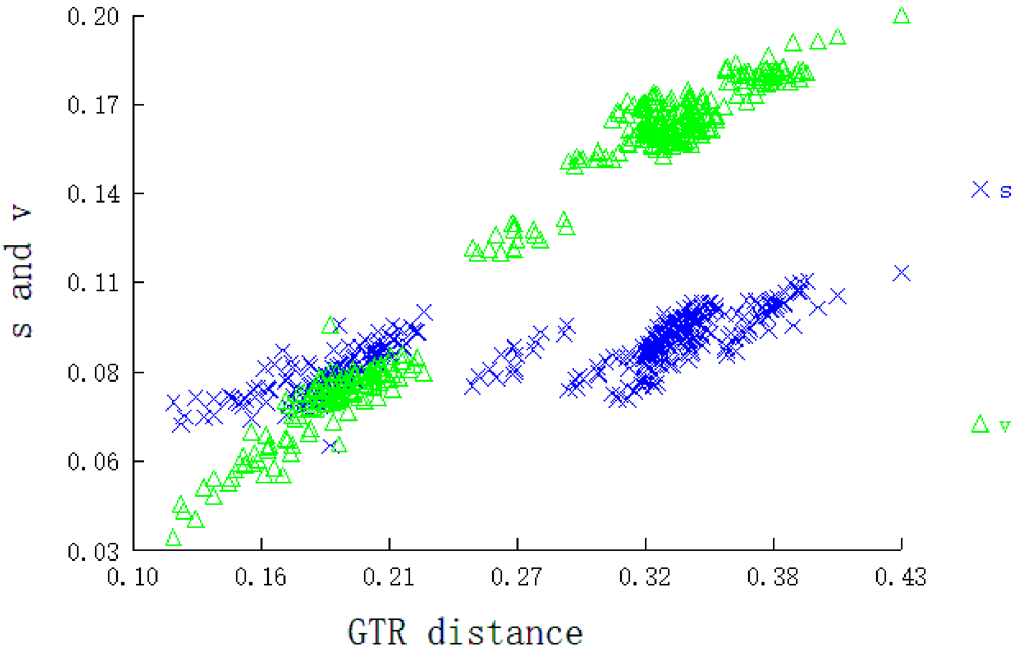

Figure S6 Substitution saturation plots of PCG123 datasets. Plots in blue and green indicate transition and transversion, respectively.

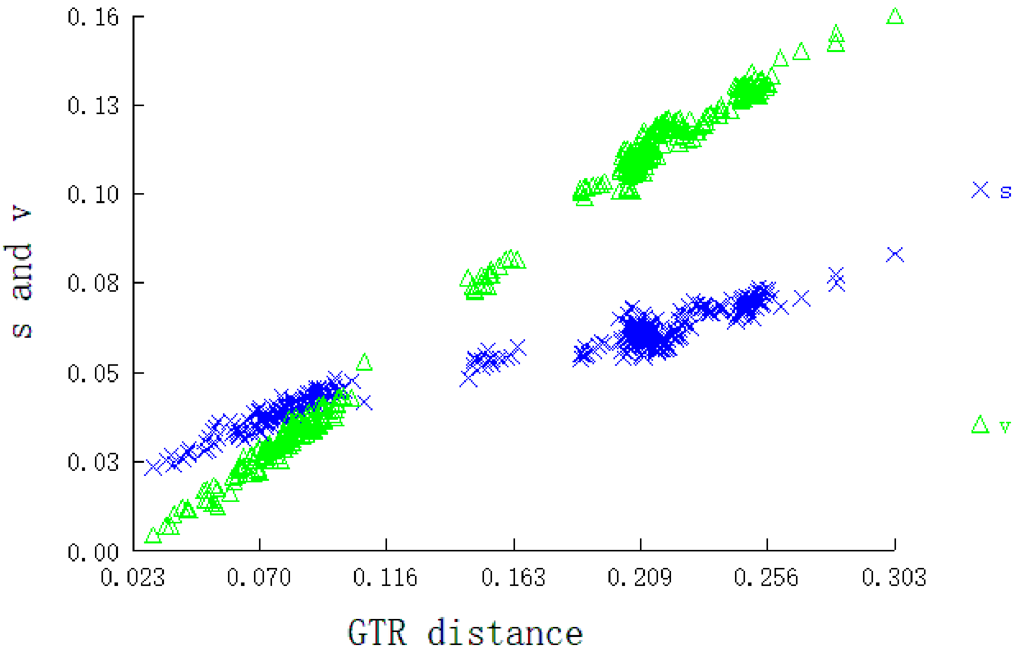

Figure S7 Substitution saturation plots of PCG12 datasets. Plots in blue and green indicate transition and transversion, respectively.

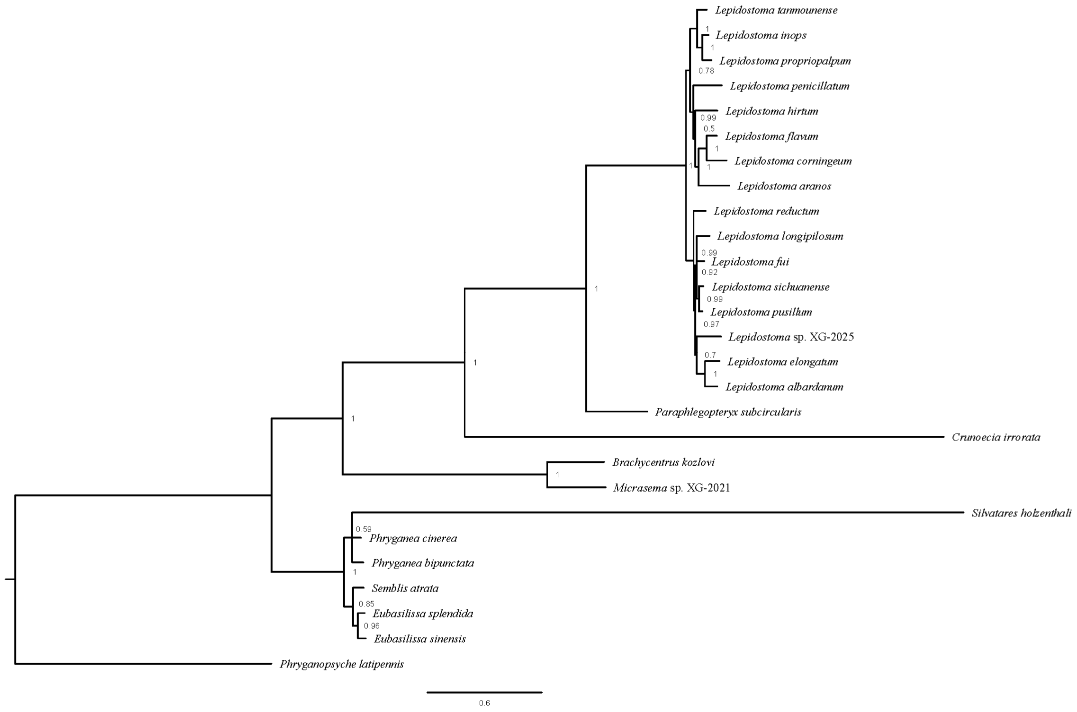

Figure S8. The BI tree based on the PCG12R dataset, using the CAT + GTR model. The numbers above nodes are Bayesian posterior probabilities.

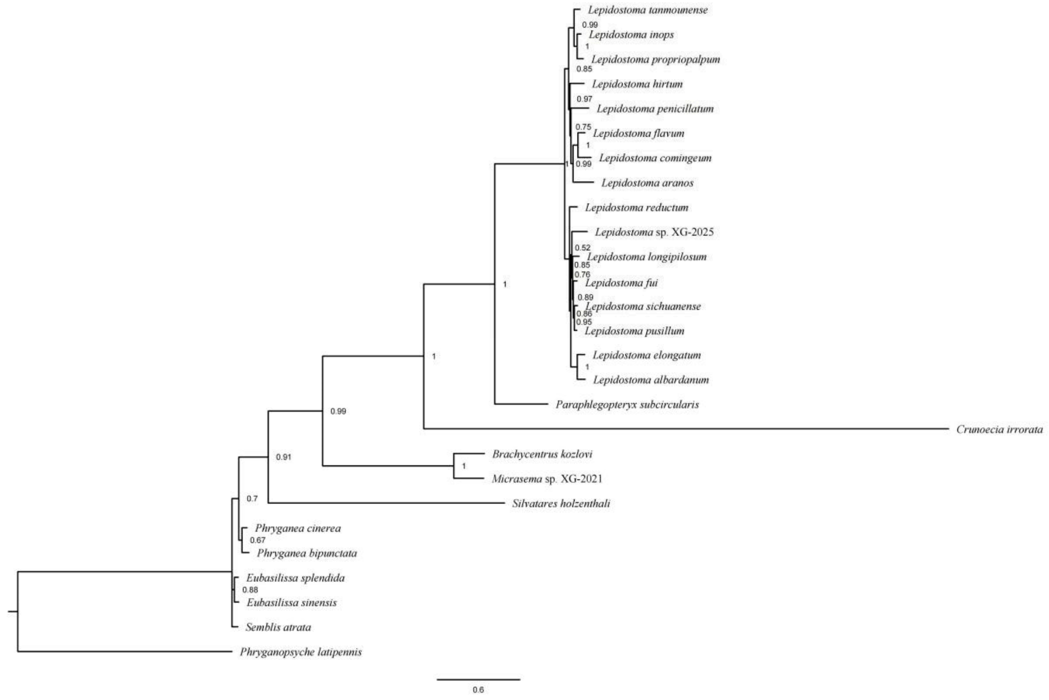

Figure S9. The BI tree based on the PCG12 dataset, using the CAT+GTR model. The numbers above nodes are Bayesian posterior probabilities.

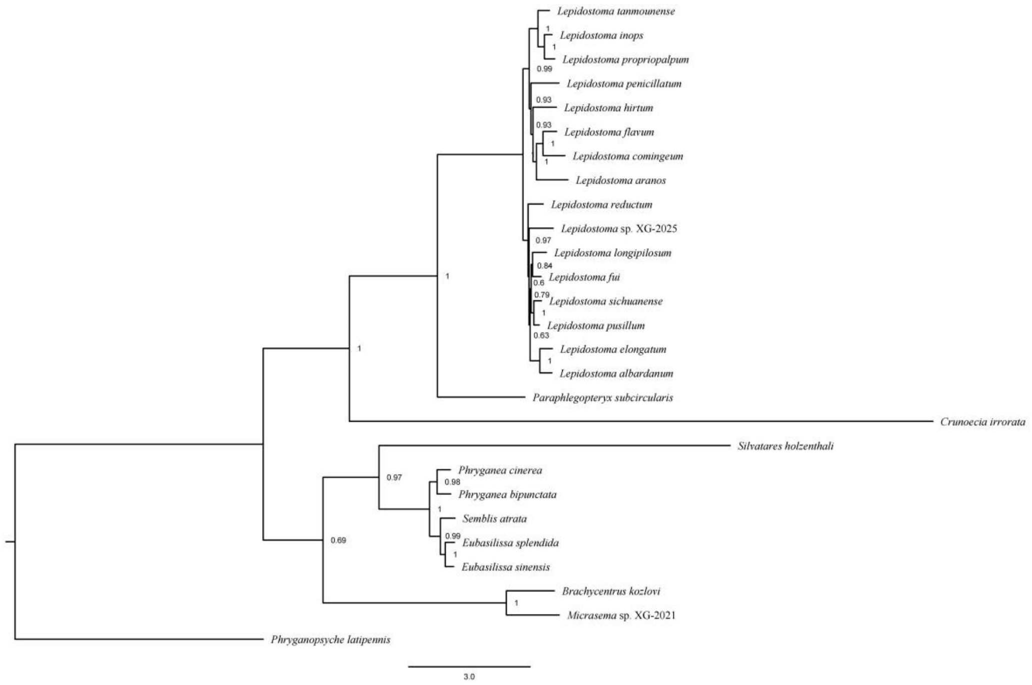

Figure S10. The BI tree based on the PCG dataset, using the CAT + GTR model. The numbers above nodes are Bayesian posterior probabilities.

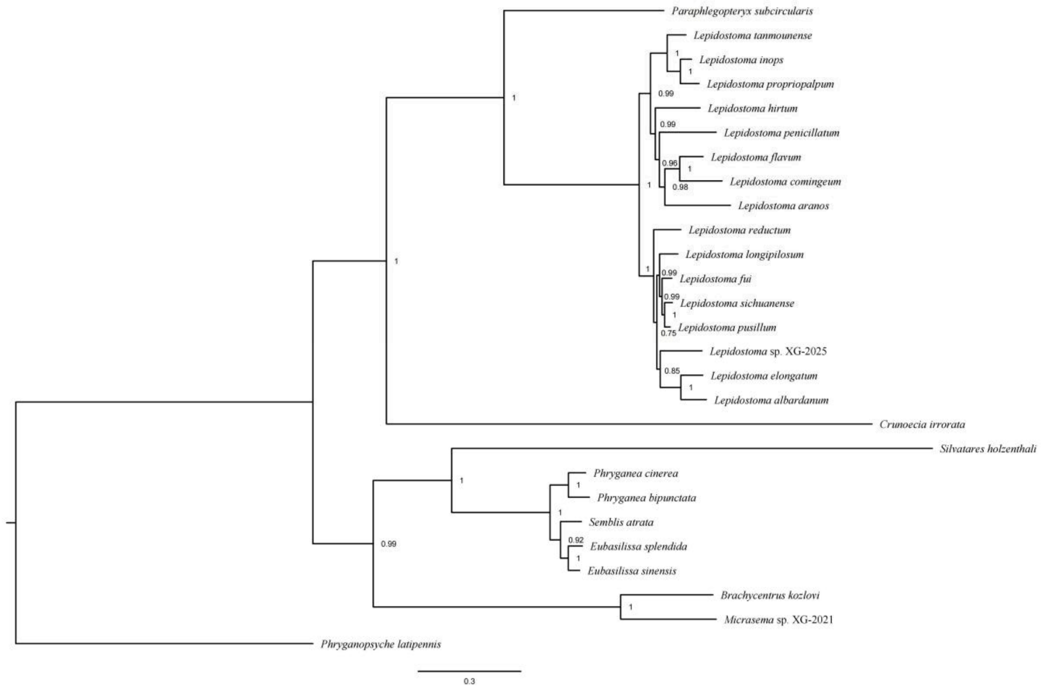

Figure S11. The BI tree based on the AA matrix dataset, using the CAT + GTR model. The numbers above nodes are Bayesian posterior probabilities.

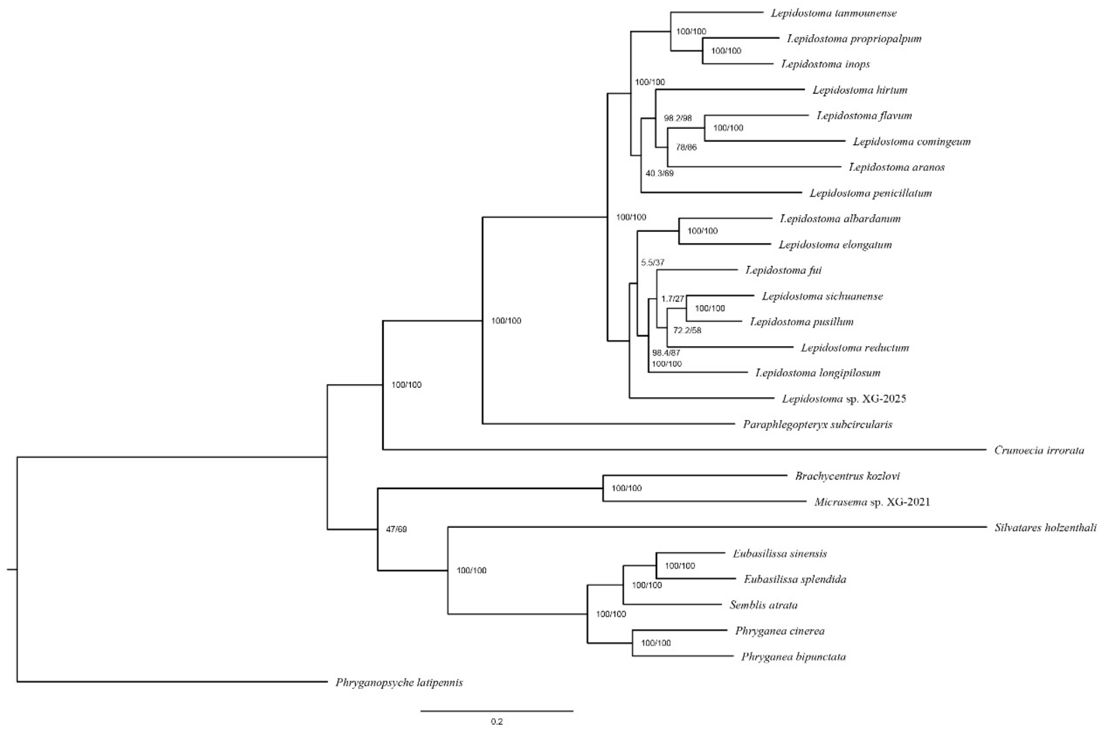

Figure S12. The ML tree based on the PCG123R dataset, using the Partitioning model. The numbers above nodes are bootstrap probabilities.

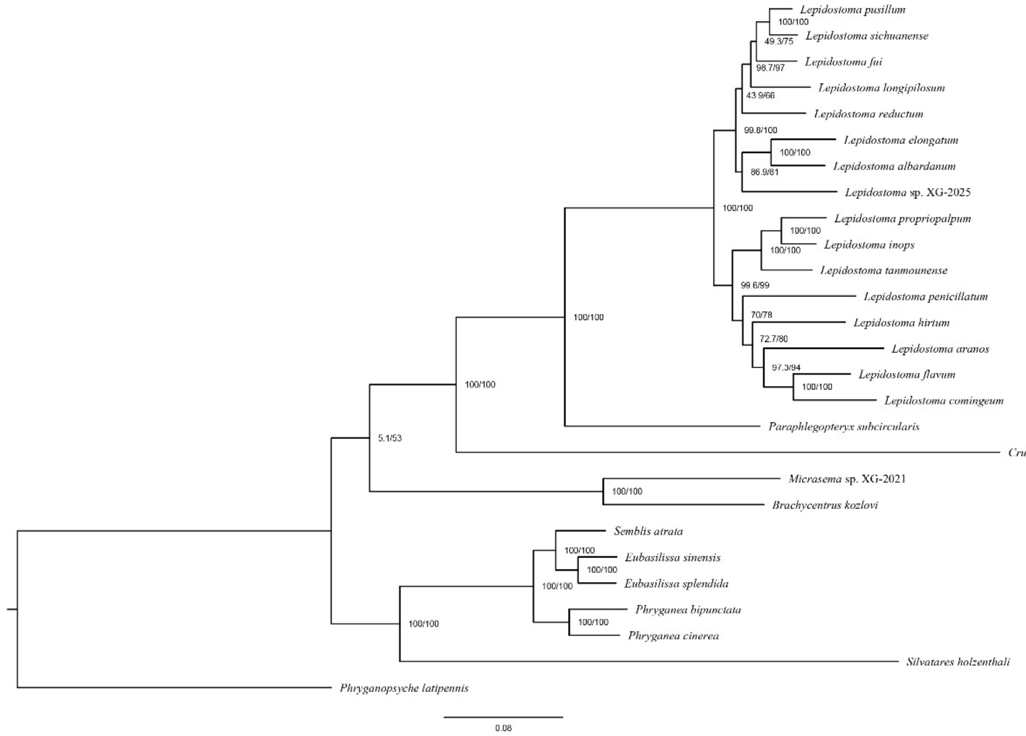

Figure S13. The ML tree based on the PCG12R dataset, using the Partitioning model. The numbers above nodes are bootstrap probabilities.

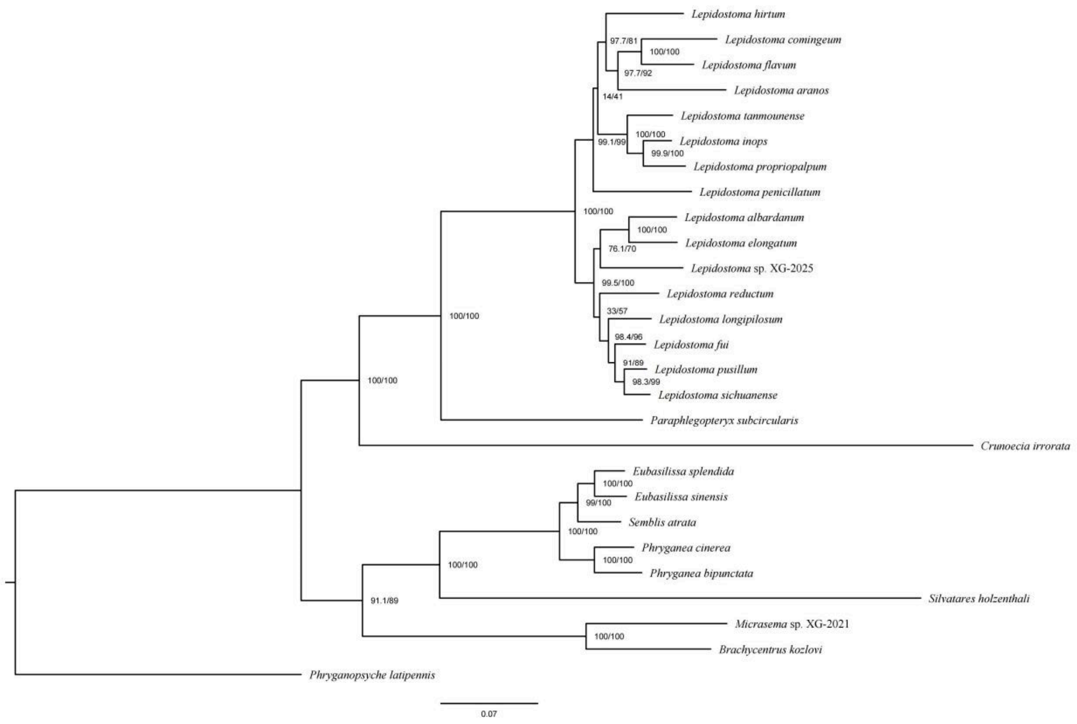

Figure S14. The ML tree based on the PCG12 dataset, using the Partitioning model. The numbers above nodes are bootstrap probabilities.

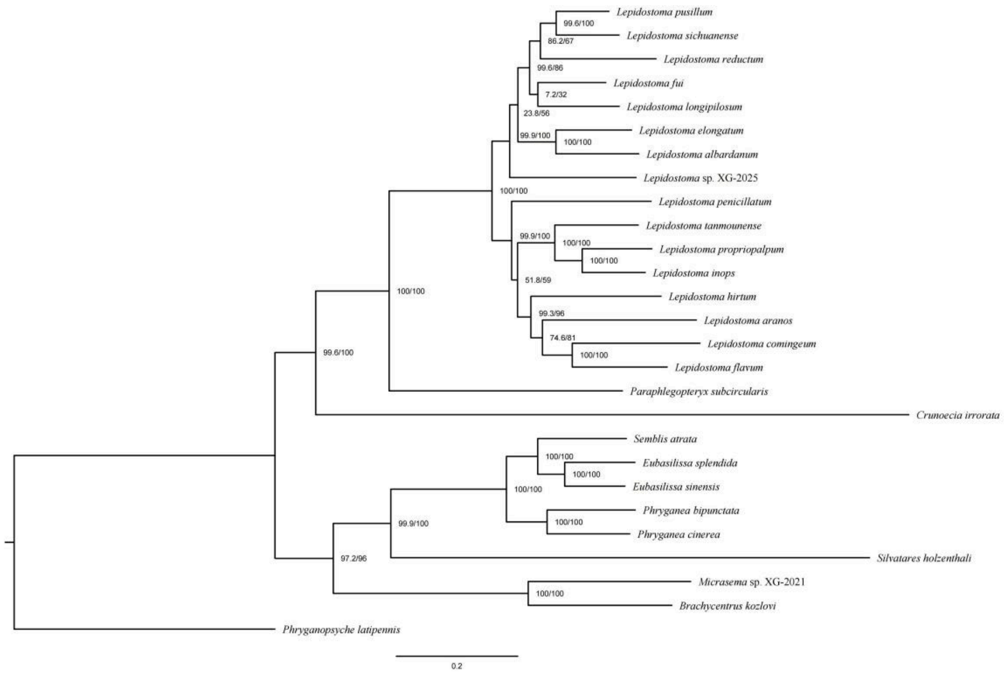

Figure S15. The ML tree based on the PCG dataset, using the Partitioning model. The numbers above nodes are bootstrap probabilities.

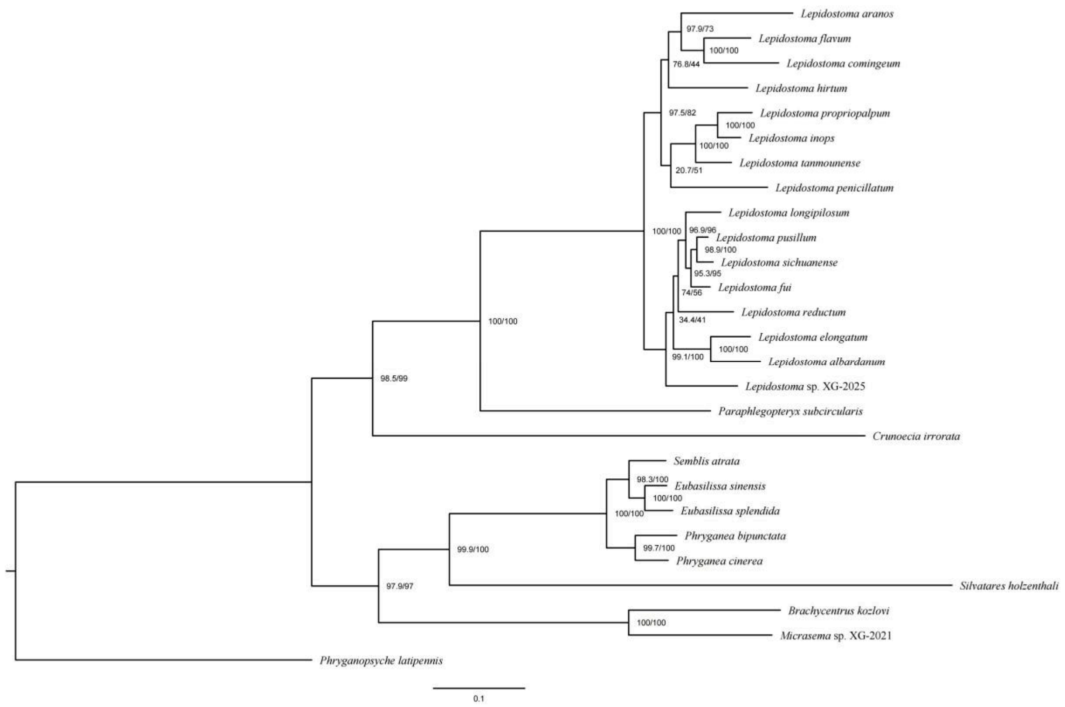

Figure S16. The ML tree based on the AA dataset, using the Partitioning model. The numbers above nodes are bootstrap probabilities.

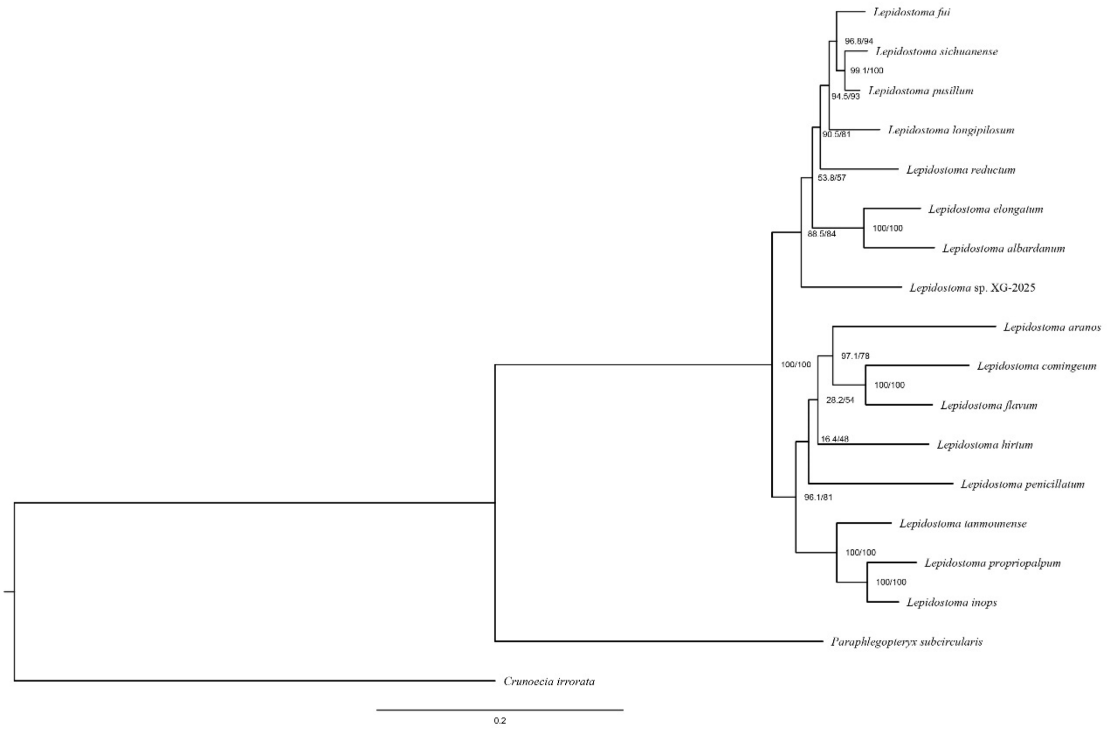

Figure S17. The ML tree based on the AA dataset of Lepidostomatidae, using the Partitioning model. The numbers above nodes are bootstrap probabilities.

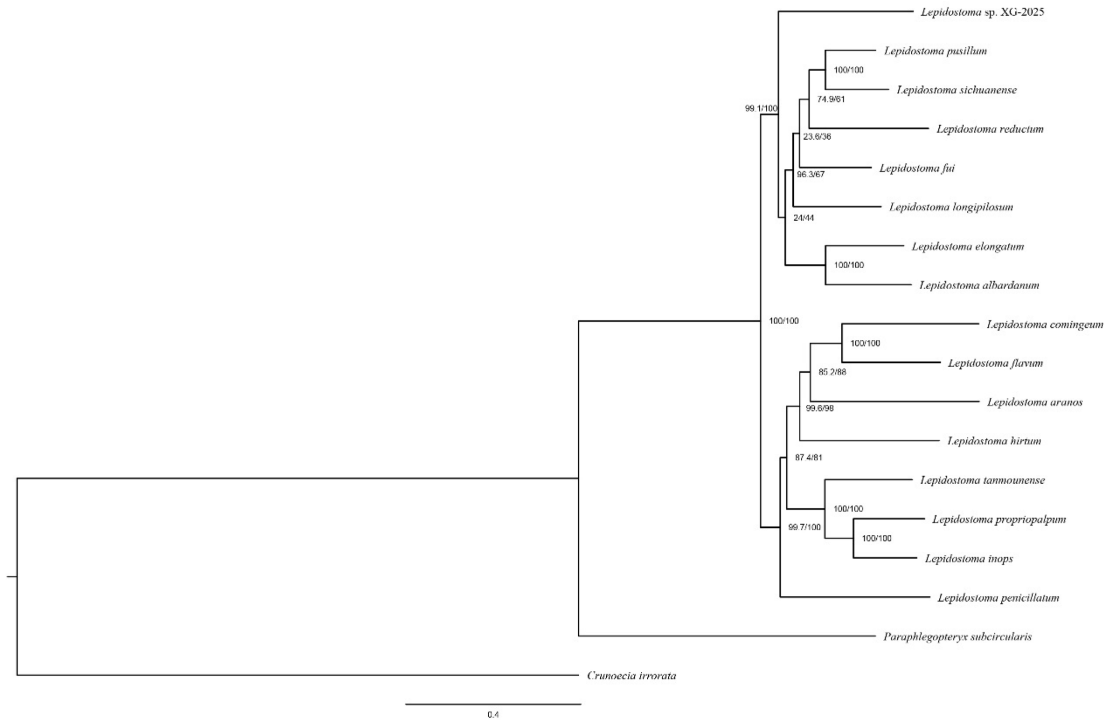

Figure S18. The ML tree based on the PCG dataset of Lepidostomatidae, using the Partitioning model. The numbers above nodes are bootstrap probabilities.

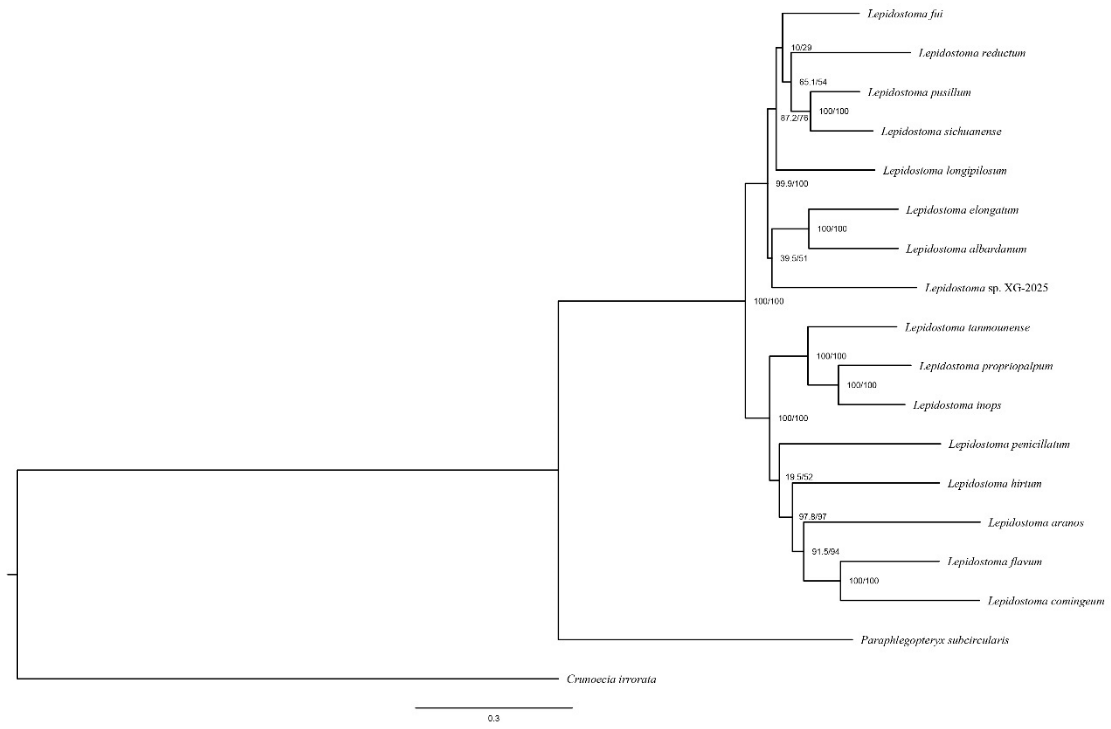

Figure S19. The ML tree based on the PCG123R dataset of Lepidostomatidae, using the Partitioning model. The numbers above nodes are bootstrap probabilities.

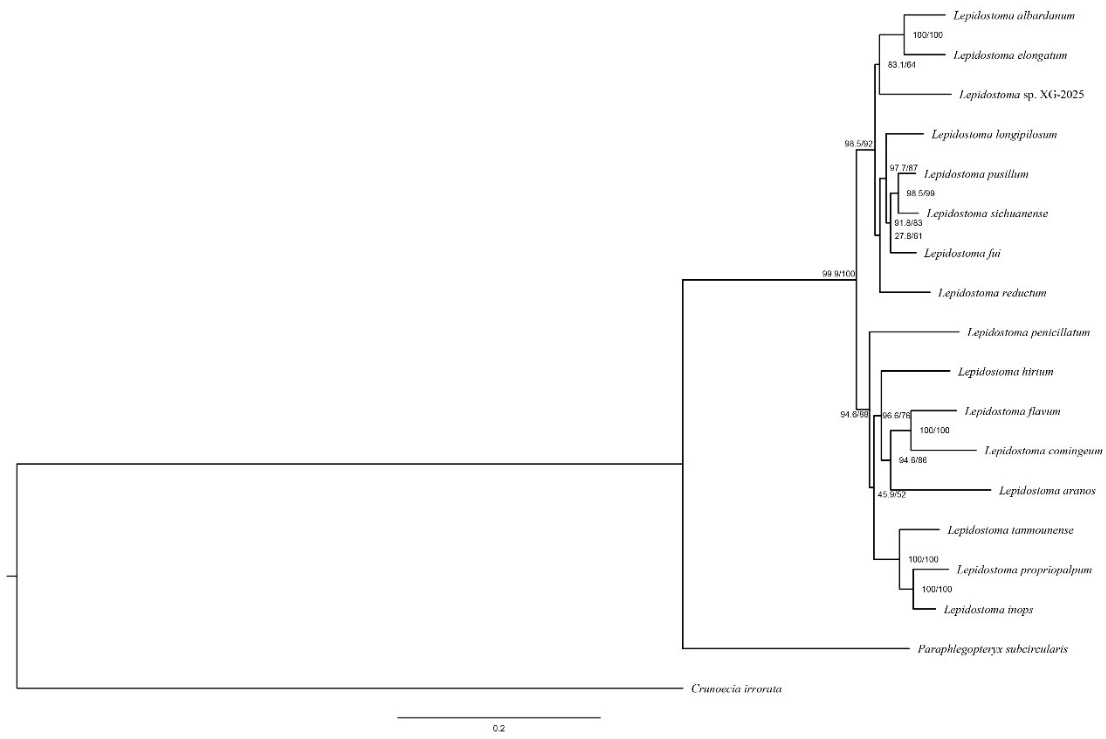

Figure S20. The ML tree based on the PCG12 dataset of Lepidostomatidae, using the Partitioning model. The numbers above nodes are bootstrap probabilities.

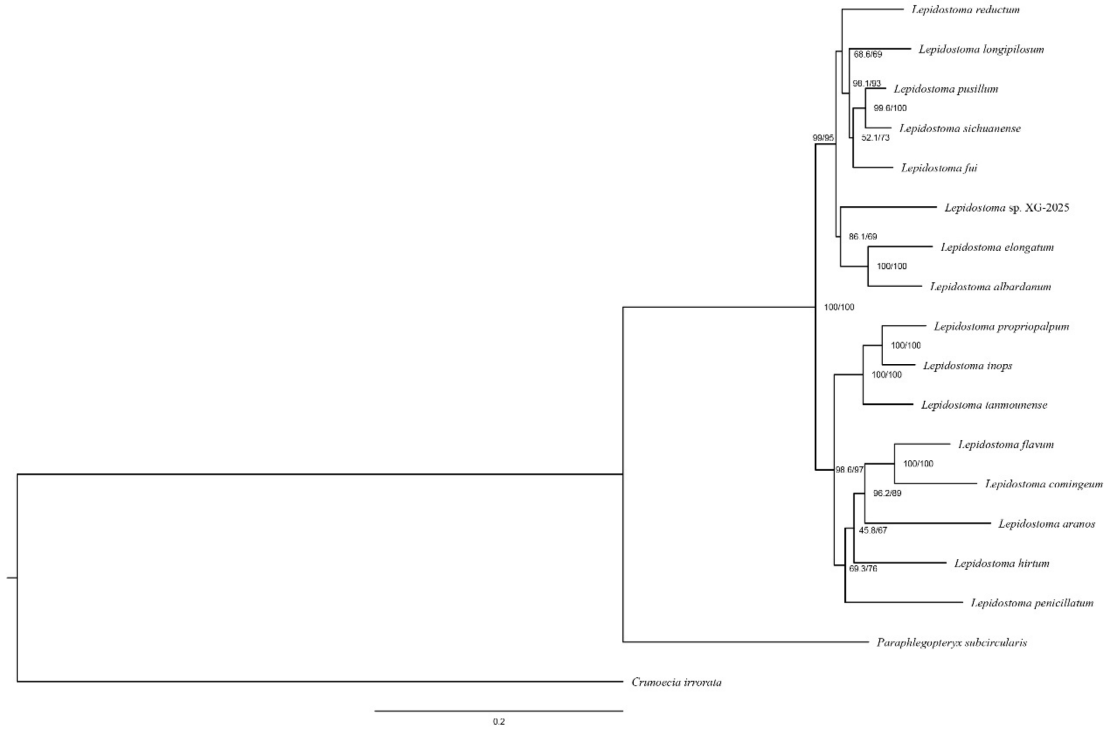

Figure S21. The ML tree based on the PCG12R dataset of Lepidostomatidae, using the Partitioning model. The numbers above nodes are bootstrap probabilities.
